# Enhancer control of promoter activity and variability via frequency modulation of clustered transcriptional bursts

**DOI:** 10.1038/s41588-026-02676-x

**Published:** 2026-07-14

**Authors:** Jana Tünnermann, Gregory Roth, Julie Cramard, Luca Giorgetti

**Affiliations:** 1 Friedrich Miescher Institute for Biomedical Research, Basel, Switzerland; 2 University of Basel, Basel, Switzerland

**Keywords:** Molecular biology, Biophysics

## Abstract

Gene expression in mammalian cells is controlled by enhancers that are often dispersed across large *cis*-regulatory landscapes around a promoter. How enhancers determine transcription of their target genes and how this depends on their relative position within a *cis*-regulatory landscape remain unclear. Here we use live-cell imaging to track the activity of a promoter under the control of the same enhancer, but inserted at different positions across a simplified regulatory landscape with minimal complexity. Combined with mathematical modeling, this reveals that RNA production from the promoter occurs in clusters of transcriptional bursts, with enhancer position controlling the frequency at which clusters appear. This results in bursts being more frequent and occurring more uniformly across cells when the enhancer is genomically close to the promoter than when it is located at a large genomic distance. Mathematical modeling further indicates that the enhancer modulates the promoter’s ability to transition from its basal transcriptional state to a regime in which clusters of bursts become more frequent. Our results reveal unexplored modes of mammalian promoter operation and show that enhancer position within a *cis*-regulatory landscape critically controls the timing and variability of transcriptional output in single cells.

## Abstract (web summary)

Tünnermann et al. use live-cell imaging to study promoter activity under the control of an enhancer inserted at different genomic distances. RNA production from the promoter occurs in clusters of transcriptional bursts whose frequency is dictated by enhancer distance.

## Main

In metazoans, transcriptional control of large numbers of genes critically relies on enhancers^1^, which are often dispersed across *cis-*regulatory landscapes spanning several hundred kilobases around their target promoters. Despite their central role in gene regulation, how enhancers control their target genes remains elusive. Studies based on population-averaged chromosome conformation capture and RNA quantification methods have suggested that enhancers communicate regulatory information to their promoters via direct physical proximity enabled by the three-dimensional (3D) folding of the chromatin fiber^2–5^. However, the spatiotemporal relationship between such encounters and RNA synthesis in single cells remains unclear, especially in mammalian cells^6^.

Inside single cells, regulatory events at enhancers and promoters are inherently probabilistic, as stochastic binding and unbinding of small numbers of transcription factors and cofactors cause discontinuous loading and licensing of RNA polymerase II (Pol II) complexes^7^. This generates intermittent bursts of promoter activity during which Pol II enters productive elongation, separated by inactive periods with no transcription^8^. Physical distances between enhancers and promoters also likely fluctuate over time^9–11^, likely restricting molecular interactions at the enhancer–promoter interface to limited time windows. Yet how promoter burst kinetics are regulated by stochastic encounters with an enhancer and how this depends on the genomic location of the enhancer within the *cis*-regulatory landscape remains poorly understood^12^. Understanding this is essential to explain how transcription levels and their cell-to-cell and temporal variability are encoded by genomic sequence.

We and others recently showed that, at the population level, promoter transcription levels increase as the genomic distance to an enhancer decreases^11,13–18^. This can be explained in terms of an increase in promoter burst frequency with increased enhancer–promoter contact probabilities, which themselves increase with decreasing genomic distance^13^, in agreement with previous observations that enhancers mainly control burst frequency^19–21^. Thus, in addition to enhancer and promoter sequences themselves, their location within a *cis*-regulatory landscape and, notably, their reciprocal genomic distance not only control average transcription levels but also impact the heterogeneity of mRNA production in single cells and thus the precision with which regulatory inputs are translated into transcriptional outputs. However, direct evidence that the large-scale organization of a genomic locus impacts burst dynamics in single cells is missing.

Recent studies have also questioned the validity of interpreting mammalian promoter burst kinetics using simplistic two-state (On/Off) models of gene expression. A growing body of work instead supports three-state models in which promoters transition between a repressive state (‘deep Off’) and a permissive state (Off), from which they can enter an active state (On) that supports RNA production^20,22,23^. Some studies have supported the hypothesis that mammalian enhancers modulate transitions either between Off states or from the permissive Off state to the On state^20,22^. Yet whether enhancers and promoters generally follow such three-state kinetics and which molecular mechanisms could account for this behavior remain unknown^24,25^.

Here we use a reductionist approach in mouse embryonic stem cells (mESCs) to study the transcriptional dynamics of the *Sox2* promoter while varying its genomic distance from its cognate Sox2 control region (SCR) enhancer within a controlled genomic region that minimizes regulatory and structural confounding effects. This allows us to isolate the effect of enhancer–promoter distance and hence contact probability, which inversely scales with genomic distance, while keeping all other regulatory variables (sequence identity, biochemical composition of the enhancer–promoter interface) constant.

Quantitative analysis of live-cell imaging shows that the genomic distance between the SCR enhancer and the *Sox2* promoter modulates burst frequency without affecting burst duration or size. Transcription occurs in clusters of short bursts separated by longer inactive periods, independently of enhancer distance. Notably, consecutive inactive periods are positively correlated, a feature not explained by standard two-state or three-state models. Mathematical modeling indicates that the promoter stochastically switches between two regimes with low and high burst frequencies, with enhancer–promoter distance modulating the transition rate from the low-frequency to high-frequency regime. This suggests that SCR encounters drive the promoter into a more transcriptionally active regime. Finally, larger enhancer–promoter distances increase cell-to-cell variability in burst timing and number, indicating that large-scale locus organization contributes to transcriptional precision in space and time.

## Results

**Fig. 1:**
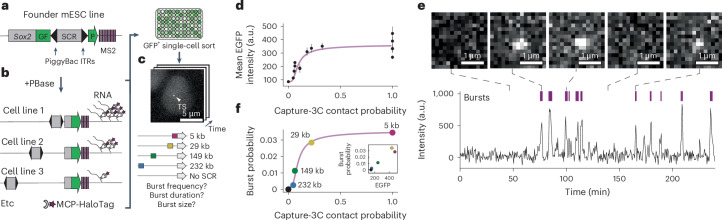
Enhancer–promoter genomic distance dictates the probability of observing transcription. **a**, Schematic of piggyBac-based enhancer (E) mobilization—the *Sox2* promoter (P) drives transcription of an *EGFP* gene split by a piggyBac–SCR cassette. The 3’ UTR of the *EGFP* transcript carries 24 MS2 stem-loop repeats. **b**, Upon expression of PBase, the enhancer is excised and reintegrated elsewhere in the genome, reconstituting the *EGFP* transgene and enabling EGFP^+^ cells to be sorted into clonal cell lines. **c**, Live-cell imaging is used to detect TS and measure burst parameters as a function of enhancer–promoter genomic distance (scale bar = 5 µm). **d**, Mean EGFP intensities assessed by flow cytometry in individual EGFP^+^ cell lines as a function of enhancer–promoter contact probability, as measured by Capture-3C. Purple trend line from ref. ^13^. Mean ± s.d., *n* = 3 measurements on different days. **e**, MS2 intensity trace from one cell with example images of TS signals (scale bar = 1 µm). Bursts were defined based on a spot being detected by the spot detection algorithm. **f**, Time-averaged probability of observing a burst at a given time point as a function of contact probability measured in Capture-3C. Purple trend from ref. ^13^. Inset—EGFP levels plotted against the corresponding probability of observing a burst. ITR, inverted terminal repeats; TS, transcription sites. Source data

To examine promoter transcriptional dynamics under the influence of a cognate enhancer with minimal confounding effects, we used an enhancer mobilization assay previously developed in mouse mESCs. This assay uses a piggyBac transposon cassette to mobilize the enhancer and reinsert it at multiple random positions around an initial site within an ectopic, neutral genomic region^13^. In this assay, the mouse *Sox2* promoter drives expression of a split enhanced green fluorescent protein (EGFP) transcript containing a piggyBac transposon cassette harboring the SCR enhancer^26^. This construct is integrated in a ‘neutral’ topologically associated domain (TAD) on chromosome 15, which is devoid of other active enhancers and promoters, active or repressive chromatin marks, or CTCF loops. To allow the visualization of RNA produced by this construct, we have integrated 24 MS2 stem-loop repeats^27,28^ in the 3’ untranslated region (UTR) of the split EGFP transgene (Fig. 1a). When transcribed, these loops can be visualized upon binding of MS2 coat protein (MCP) fused to HaloTag, which was stably expressed following random integration of the corresponding expression construct. Upon expression of the PBase transposase, the enhancer transposon cassette is excised and randomly integrated into the genome (Fig. 1b). Reconstitution of a functional *EGFP* transcript followed by sorting of EGFP^+^ cells enables the generation of clonal mESC lines, in each of which the SCR is located at a different position within the ‘neutral’ TAD. This approach ensures that all clonal mESC lines exhibit nearly identical MCP-HaloTag expression levels, with enhancer–promoter genomic distance as the only variable, enabling direct comparison of live-cell imaging data, transcription start site detection and burst parameters such as frequency, duration and size across clones (Fig. 1c)

**Extended Data Fig. 1:**
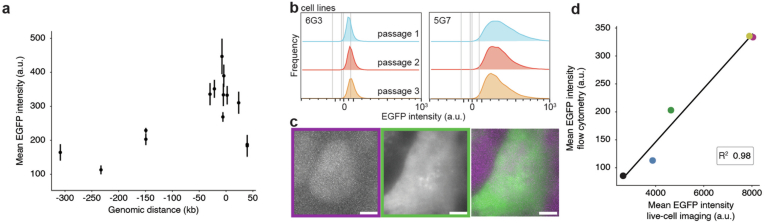
EGFP levels measured in live-cell imaging experiments and flow cytometry are correlated. **a**, EGFP levels of individual EGFP+ cell lines over cell passages measured by flow cytometry. Mean ± s.d. *n* = 3 measurements on different days. **b**, Mean EGFP intensity assessed through flow cytometry in individual EGFP+ cell lines as a function of genomic distance between the promoter and the enhancer. **c**, Representative images from live-cell imaging of the MCP-HaloTag channel (640 nm laser, left), EGFP channel (480 nm laser, middle) and overlay (right). Scale bar: 5 µm. One representative cell is shown with the enhancer located at −5 kb. **d**, Pearson correlation of mean EGFP levels measured using flow cytometry (mean of *n* = 3 measurements on different days) with mean EGFP levels measured in live-cell imaging (mean over all cells imaged for each clonal line; sample sizes: from 146’798 to 240’303 cells per sample, depending on clonal line). Pearson coefficient of determination (r2) = 0.98. coefficient of determination (r2) = 0.98. Source data

Using this modified enhancer mobilization assay, we generated 14 clonal mESC lines with genomic distances ranging from 5 to over 300 kb (Extended Data Fig. 1a), plus one in which the SCR was reintegrated on a different chromosome where it exerts no effect on the ectopic *Sox2* promoter (Supplementary Table 1). In these lines, population-averaged EGFP protein levels assessed by flow cytometry remained stable over passages (Extended Data Fig. 1b). EGFP levels decreased with increasing enhancer–promoter genomic distance (Extended Data Fig. 1a) and scaled nonlinearly with the contact probability between enhancer and promoter positions, as inferred from prior Capture-C data, which at this locus is largely unaffected by the presence of the enhancer and promoter themselves^13^ (Fig. 1d). This trend is well described by the nonlinear relationship that we previously measured between EGFP expression levels and contact probabilities using the same assay in the absence of MS2 stem loops (Fig. 1d), indicating that the stem loops do not perturb transcriptional behavior of the *Sox2* promoter under the control of the SCR.

**Extended Data Fig. 2:**
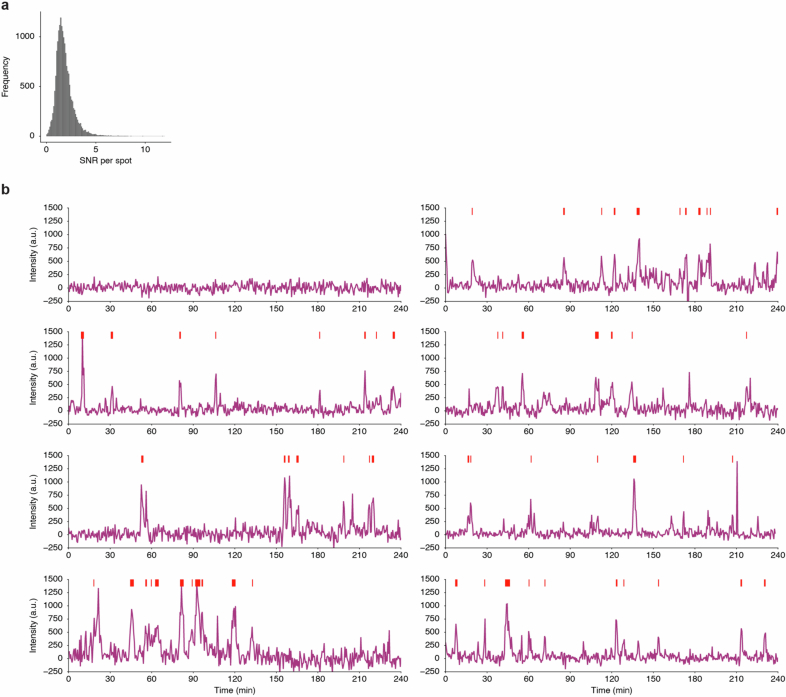
Signal-to-noise ratio and representative MS2 intensity traces. **a**, Histogram of the signal-to-noise ratio (SNR) per spot, defined as the difference between spot intensity and local background intensity divided by the standard deviation of the local background. **b**, Examples of bleach- and background-corrected MS2 intensity traces. Red markers show bursts that were defined based on a spot being detected by the spot detection algorithm. Source data

We then selected a subset of five clonal mESC lines in which the enhancer was reintegrated at genomic distances of 5, 29, 149, 232 kb (Fig. 1c) or on a different chromosome for further analysis. In each line, we acquired one EGFP 10-µm *z* stack followed by movies of 10-µm *z* stacks every 30 s for 5 h using oblique illumination microscopy (Supplementary Videos 1 and 2). Average EGFP levels measured by microscopy strongly correlated with those obtained by flow cytometry, indicating that the imaged cells were representative of the overall population (Extended Data Fig. 1c,d). We then quantified the transcriptional kinetics by detecting transcription sites as diffraction-limited spots (mean signal-to-noise ratio (SNR) = 1.8; Extended Data Fig. 2a) in time-resolved image series. Transcriptional bursts were defined as consecutive time frames in which a transcription start site was detected (Methods). Imaging of three to seven technical replicates per condition yielded between 1,000 and 2,500 MS2 intensity tracks per condition, with varying durations (example traces in Fig. 1e and Extended Data Fig. 2b, and summary of track statistics in Supplementary Table 2). Time-averaged probabilities of observing a burst at a given time point correlated strongly with EGFP levels and revealed the same nonlinear relationship as previously reported for transcriptional levels measured by mature mRNA or protein^13^ (Fig. 1f), suggesting that the SCR predominantly controls the probability of initiating a burst from the *Sox2* promoter.

### Enhancer–promoter genomic distance affects interburst duration

**Extended Data Fig. 3:**
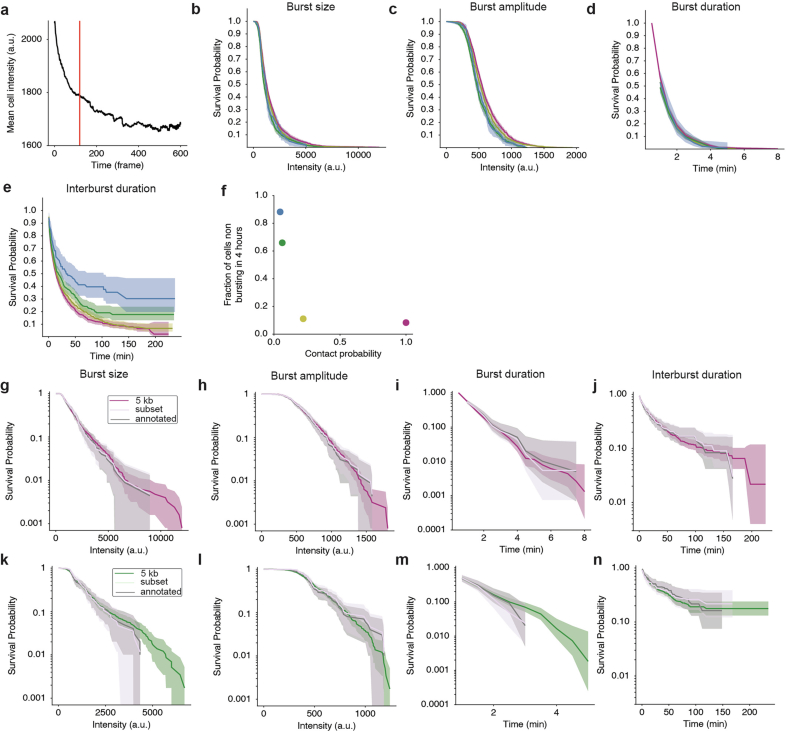
Quantification of burst parameters is representative of the underlying dynamics. **a**, Average intensity of nuclear background as a function of frame number. Red line: lower bound (120 frames), the time window considered in the burst analysis. **b**–**e**, Experimental data are the same as in Fig. 2a–d, but shown in a linear scale. **f**, Fraction of non-bursting cells as function of contact probability as estimated by Capture 3C^13^. **g**–**n**, Comparison of burst parameters between the full dataset (colored line), a subset of the dataset (dark gray line) and manually annotated versions of the same subset (light gray line) for two clonal cell lines (upper panel = 5 kb enhancer-promoter genomic distance, lower panel = 149 kb enhancer-promoter genomic distance). Error bands and centers of error bands are calculated as in Fig. 2a–d. Source data

We next examined how quantitative burst parameters vary with enhancer–promoter genomic distance, and thus contact probability. To this end, we computed survival distributions of burst size, amplitude, duration and interburst intervals across the five mESC lines with distinct SCR positions. To avoid confounding effects from fluorescence levels declining over the first hour of imaging (Extended Data Fig. 3a), the first 120 frames were removed from this analysis.

**Fig. 2:**
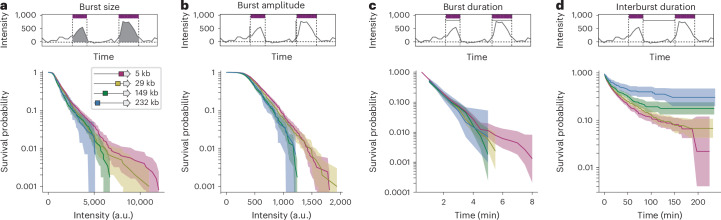
Quantification of burst parameters from live-cell imaging. Top, schematics of the quantified parameters. **a**, Survival probability of burst sizes. **b**, Survival probability of burst amplitudes. Shaded areas in **a** and **b** show pointwise 95% confidence intervals obtained by bootstrap resampling of events, defined by the 2.5th and 97.5th percentiles of the survival probability at each time point (*n* = 10,000). **c**, Survival probability of burst durations. **d**, Survival probability of interburst durations. In **c** and **d**, solid lines show the Kaplan–Meier estimate of the survival probability. Shaded areas represent approximate 95% confidence intervals centered on these estimates, computed using Greenwood’s s.e. on the log scale (±2 × s.e., pointwise). Source data

**Fig. 3:**
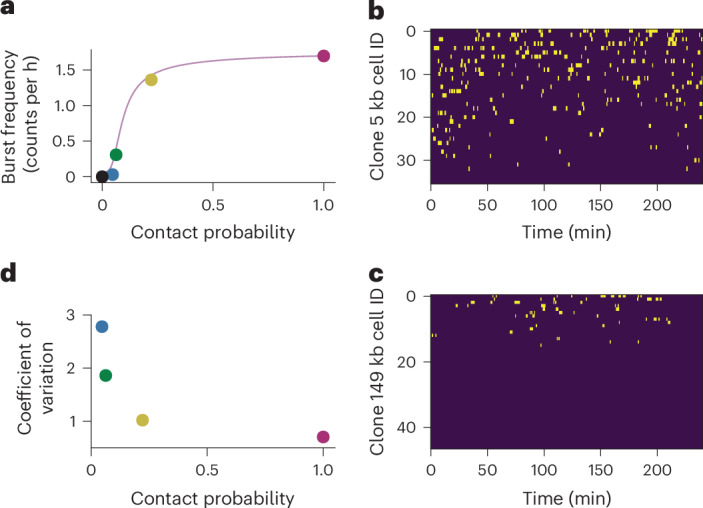
Enhancer–promoter genomic distance modulates burst frequency and its variability across cells. **a**, Burst frequency (number of bursts per hour) as a function of enhancer–promoter contact probability (mean over 4-h tracks). Purple trend line from ref. ^13^. **b**,**c**, Binarized intensity traces during at least 4 h for clonal cell lines with 5 kb (**b**) and 149 kb (**c**) enhancer–promoter genomic distance. Each row corresponds to one cell; traces are sorted by the number of bursts in the trace. **d**, Cell-to-cell variability in burst frequency as a function of contact probability, quantified via the coefficient of variation of burst frequencies. Source data

Burst size and amplitude, defined as the integrated and peak MS2 signal during active transcription, showed no substantial variation across enhancer–promoter distances, as their survival distributions remained within the 95% confidence interval (CI) estimated by bootstrapping (10,000 repeats; Fig. 2a,b and Methods). We observed only a slight decrease in burst amplitude with increasing enhancer–promoter genomic distance (Fig. 3a,b and Extended Data Fig. 3b,c).

Because imaging was performed over finite time windows, some active or inactive periods were censored, initiating or terminating outside the observation window. To account for this, we estimated burst duration and interburst interval distributions using the Kaplan–Meier estimator (Methods). Censoring-corrected burst durations were independent of enhancer–promoter genomic distance (Fig. 2c and Extended Data Fig. 3d), with individual bursts being relatively short, exhibiting a median survival time of approximately 1.5 min. In contrast, interburst durations increased with enhancer–promoter genomic distance (Fig. 2d and Extended Data Fig. 3e), with median values rising from ~15 min at short distances (5–30 kb) to ~30 min at 232 kb and broad distributions spanning from minutes to several hours. Interestingly, the probability that interburst intervals lasted more than 4 h increased with increasing enhancer–promoter distance, ranging from 2% at 5 kb to 30% at 232 kb (Fig. 2d). This reflects the observation that the fraction of cells that never burst in 4 h also increased with increasing distance (Extended Data Fig. 3f).

The automated image analysis pipeline achieved 97% precision and 57% recall (F1 = 0.72) relative to manual annotations (one movie per clonal line). To verify that burst measurements were not artifacts, we manually curated 10% of the data for clones with enhancer–promoter distances of 5 kb and 149 kb. The resulting survival distributions were indistinguishable, validating the analysis pipeline (Extended Data Fig. 3g–n).

Together, these data indicate that genomic distance to the SCR enhancer modulates burst frequency of the *Sox2* promoter, with no effect on burst duration and only very minor effects on burst amplitude and size.

### Burst frequency and its variability across cells scale nonlinearly with contact probability

Consistent with these findings, the *Sox2* promoter’s burst frequency, defined as the average number of active transcription periods per hour, recapitulated the nonlinear relationship between transcriptional output and contact probability observed previously^13^ (Figs. 1d,f and 3a). As previously noted, however (Fig. 2d), interburst durations are highly variable within each clone. To illustrate this, we examined two clones with enhancer–promoter distances of 5 kb and 149 kb, displaying average burst frequencies of ~7 and ~1 burst per 4 h, respectively. Binarized single-cell traces revealed substantial variability—at 5 kb, many cells exhibited far more than seven bursts per hour, whereas at 149 kb, many cells showed no bursts at all (Fig. 3b,c). Quantification of this effect using the coefficient of variation of burst frequency showed that genomic proximity of the enhancer to the promoter correlates with reduced cell-to-cell variability in the number of bursts produced over a defined amount of time (Fig. 3d). This indicates that enhancer–promoter genomic distance controls the reliability of promoter output in time.

### Transcriptional bursts occur in clusters

**Fig. 4:**
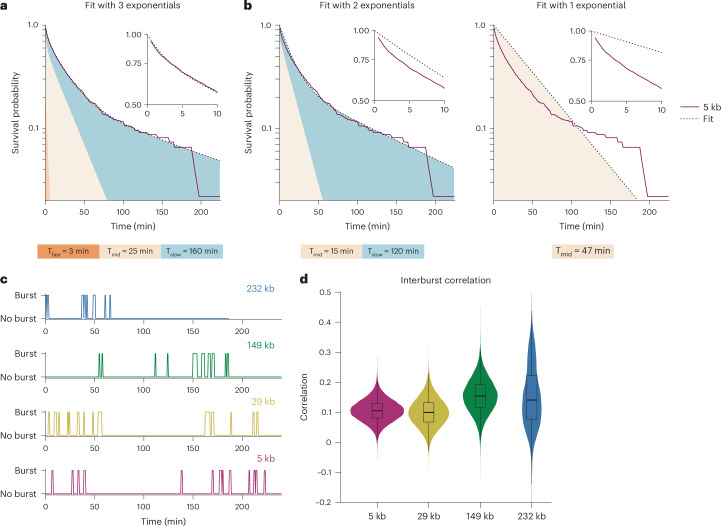
Interburst durations are distributed over three timescales. **a**, Best fit of the three-exponential model to experimental survival probabilities of the interburst durations in the cell line where the SCR is located 5 kb away from the promoter. The best-fit curve (black dotted line) is the weighted sum of three exponential curves represented by colored areas from the highest rate (orange) to the lowest rate (blue). The timescale of each exponential curve (1/rate) is shown at the bottom. Insets—zoom of the plots over the 0–10 min range. **b**, Same as **a**, but for the two-exponential model (left) and one-exponential model (right). **c**, Examples of binarized traces illustrating clusters of bursts. Color corresponds to cell line identity as in Fig. 2. **d**, Violin and box plots show the distribution of Pearson correlation values between consecutive interburst periods obtained from 40,000 bootstrap resamples of the set of tracks. Boxes indicate IQR (25th–75th percentiles), with the median shown as a horizontal line. Whiskers extend to the most extreme data points within 1.5× IQR. Source data

**Extended Data Fig. 4:**
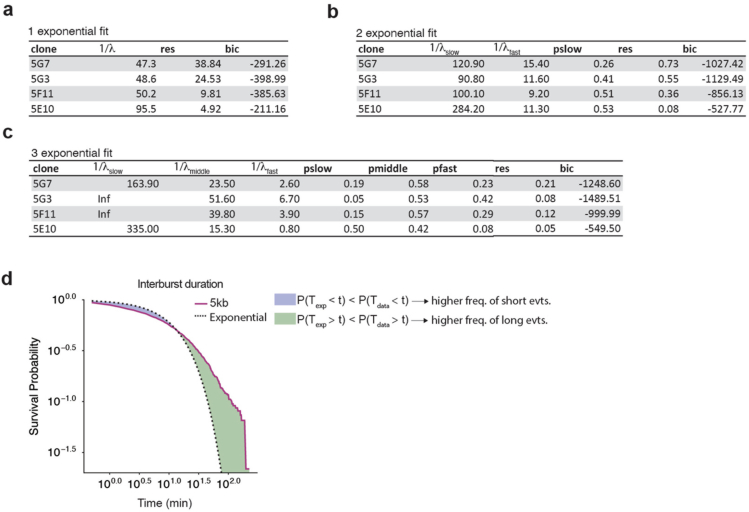
Inter-burst durations are distributed over three timescales. **a**, Parameter values for the best-fitting one-exponential model to survival probabilities of inter-burst durations in cell lines where the SCR was located at 5 kb, 29 kb, 149 kb, and 232 kb from the promoter. The inverse of the best-fit exponent is shown in minutes. The residual of the least squares fit (res) and the Bayesian information criterion (bic) are shown. **b**, Similar to panel (**a**), but for a two-exponential model. Pslow is the weight associated with the slowest exponential. **c**, Similar to panel (**a**), but for a three-exponential model. **d**, Survival probabilities of the inter-burst durations for the cell line where the SCR was located at 5 kb from the promoter (red line). Survival probabilities of an exponential variable matching the median of the inter-burst durations (black dotted line). For a time t where the survival probabilities of the inter-burst durations are below (resp. above) the survival probabilities of the exponential, the probability that events lasted less (resp. more) than a time t is higher in the data than for a Poisson process (blue and green areas, resp.). Source data

Contrary to predictions of a simple two-state model, interburst survival distributions (Fig. 2d) were not single exponentials but were best described by a weighted sum of three exponential components (Fig. 4a and Extended Data Fig. 4c). The lifetimes of the exponentials varied between clones within 3–7 min, 24–52 min, and >164 min (Extended Data Fig. 4c). Fitting with one or two exponentials failed to reproduce the full behavior of the curve (Fig. 4b and Extended Data Fig. 4a,b). These multiple timescales over which no burst is observed suggest not a single Off state, but rather multiple Off states with distinct lifetimes.

Visual inspection of the tracks suggested that bursts often occur in groups of consecutive events separated by longer periods of inactivity (Fig. 4c). Consistent with this, quantitative analysis showed that interburst survival distributions are enriched for both short and long intervals relative to a single exponential (Extended Data Fig. 4d). This is a signature of clustered patterns in a stochastic sequence of events^29^, indicating that bursts from the promoter do not occur randomly but rather in clusters.

**Extended Data Fig. 5:**
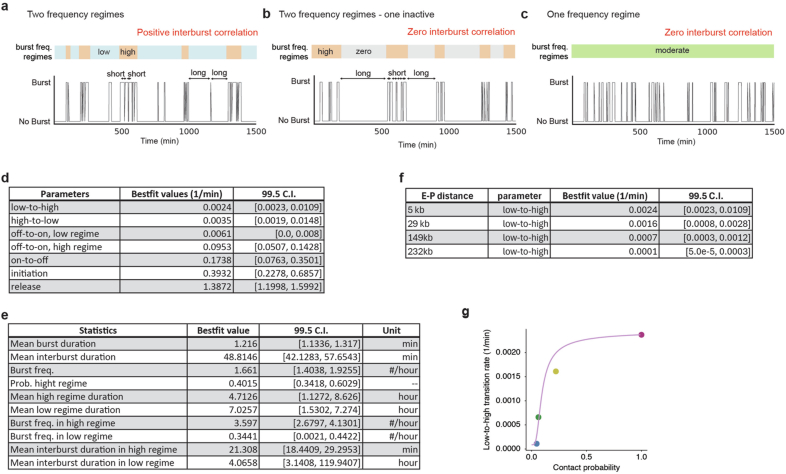
Fit with a double-regime two-state model: inferred parameters and burst statistics. **a**–**c**, Schematic of the single-cell burst dynamics under the assumption of two frequency regimes (**a**), two frequency regimes with one of them being inactive (**b**), and one frequency regime (**c**). Spearman correlation between consecutive inter-burst periods can be positive when the promoter operates in two different active regimes (**a**), but is zero when only one regime is active (**b**–**c**). **d**, Parameter values for the best-fitting double-regime, two-state model for the data of the 5 kb clone. 99.5% confidence intervals (C.I.) were calculated using a bootstrap procedure (Supplementary Note). The unit of the rates is 1/minute. **e**, Predictions of the statistics of the promoter dynamic calculated from the best-fitting model for the 5 kb clone. 99.5% confidence intervals (C.I.) were calculated using a bootstrap procedure. **f**, Values of the low-to-high regime transition rate for the best fitting double-regime, two-state model for the data of the 29, 149, 232 kb clones. All the other parameters were fixed to their best-fit value obtained for the 5 kb clone. 99.5% confidence intervals (C.I.) were calculated using a bootstrap procedure. **g**, Low-to-high regime transition rate as a function of contact probability measured in Capture-3C. Purple trend from ref. ^13^. Source data

We found a small but positive correlation between consecutive interburst intervals, such that short intervals tend to follow short ones and long intervals follow long ones (Fig. 4d). This indicates that the promoter operates in at least two regimes—a ‘high-frequency’ regime in which the promoter is frequently transcribed (short interburst periods are followed by other short interburst periods) and a ‘low-frequency’ regime in which the promoter bursts only sporadically (long interburst periods are followed by other long interburst periods; Extended Data Fig. 5a). If this were not the case—that is, if the promoter operated in a single frequency regime or in two regimes of which one never bursts—then correlations would be zero (Extended Data Fig. 5b,c). Together, these observations indicate that transcriptional dynamics in this system cannot be described by models of promoter operation that consider a single frequency regime and, notably, previously proposed two-state or three-state models with a single On state and one or multiple Off states.

### Modeling the multistate kinetics of promoter operation

**Fig. 5:**
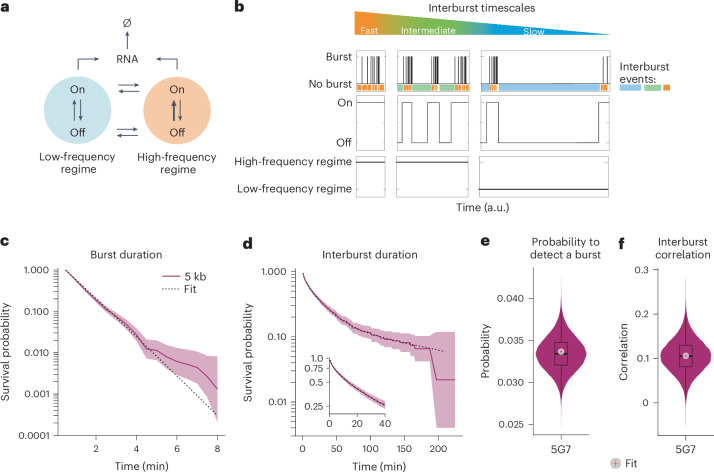
A double-regime two-state model of transcriptional dynamics. **a**, Diagram of the model. The promoter operates in a ‘low-frequency’ regime with a low On rate and can transiently enter a ‘high-frequency’ regime with a higher On rate (bold arrow). **b**, Schematics of transcriptional burst dynamics (ongoing transcription), promoter On/Off states and promoter low/high regimes, illustrating the three timescales of the interburst durations. **c**–**f**, Best fit of the dual-regime two-state model to observed survival probabilities of burst durations (**c**), interburst durations (**d**), steady-state probabilities to detect a burst (**e**) and Pearson correlation of consecutive interburst periods (**f**) for the cell line in which the SCR was located 5 kb from the promoter. In **c** and **d**, magenta solid lines show the Kaplan–Meier estimate of the survival probability. Shaded areas represent approximate 95% confidence intervals centered on this estimate, computed using Greenwood’s s.e. on the log scale (±2 × s.e., pointwise). In **e** and **f**, violin and box plots show the distribution of the probability to detect a burst (**e**) and the distribution of correlation values between consecutive interburst periods (**f**) obtained from 40,000 bootstrap resamples of the set of tracks. Boxes indicate IQR (25th–75th percentiles), with the median shown as a horizontal line. Whiskers extend to the most extreme data points within 1.5× IQR. Red crosses indicate the value predicted by the best-fit model. Source data

We next asked which stochastic model of promoter operation could account for the ensemble of our experimental observations. The simplest double-regime model consistent with positive correlations and three distinct interburst timescales is a stochastic switch between two distinct two-state regimes. In this model, each of the two regimes has an On state from which RNA synthesis can be initiated and an Off state that is not transcribed; the two regimes differ only in their Off-to-On transition rates, which are small in the ‘low-frequency’ regime and larger in the ‘high-frequency’ regime (Fig. 5a). In such a model, the longest and intermediate interburst timescales arise from Off→On transitions in the low-frequency and high-frequency regimes, respectively. The fastest timescale arises when the rate of transcription initiation in the On state(s) is low compared to the rate at which RNA is released after transcription, creating a gap in the fluorescence signal (Fig. 5b).

To test whether this model can quantitatively reproduce our observations, we first fitted it to data from a single clone (enhancer–promoter distance = 5 kb). Specifically, we simultaneously fitted the following four observables: the survival distributions of (1) burst and (2) interburst durations, (3) the steady-state probability of observing a burst and (4) the correlation between consecutive interburst intervals (Supplementary Note—Model analysis). Given the large parameter space and the computational cost of simulating all stochastic events, direct simulation-based optimization was impractical. To address this, we developed an analytical framework that derives closed-form expressions for the observables, enabling efficient parameter exploration (Supplementary Note—Model analysis). The model reproduced these four experimental observables well (Fig. 5d–f). Best agreement occurred with an average burst duration of 1.2 min (99.5% CI = 1.13–1.32), an average interburst duration of 4 h (3.1–119.9) in the low-frequency regime and 21 min (18.4–29.3) in the high-frequency regime. The high-frequency regime lasts on average 4.7 h (1.1–8.6) and produces 3.6 bursts (2.7–4.1) per hour. The low-frequency regime lasts on average 7 h (1.5–7.3) and produces 0.3 bursts (0.002–0.44) per hour. In steady state, 40% of cells (34–60) are in the high-frequency regime, resulting in an overall average interburst duration of 48.8 min (42.1–57.7) and an overall average of 1.7 (1.4–1.9) bursts per hour (full parameters in Extended Data Fig. 5d).

**Fig. 6:**
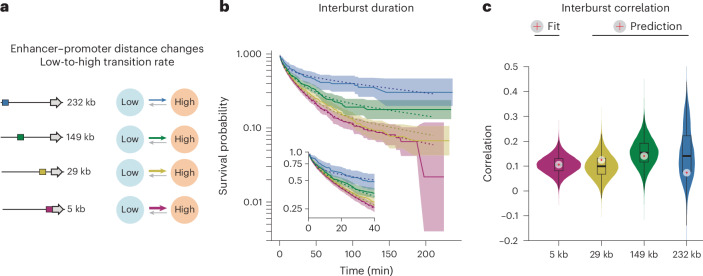
Enhancer–promoter genomic distance controls the rate of switching from the low-frequency to the high-frequency regime. **a**, Schematic of the model where enhancer-promoter distance selectively modulates the transition from the low-activity to the high-activity promoter state, while all other transition rates remain unchanged. **b**, Survival probabilities of interburst duration in cell lines with the SCR at different distances from the promoter can be predicted (gold, green and blue dotted lines) from the model fitted to the cell line in which the SCR was located at 5 kb (magenta dotted line), with a modified low-to-high regime rate. Data are similar to Fig. 2d. **c**, Red crosses show best-fit values and predictions of the Pearson correlation between consecutive interburst periods. Experimental data are the same as in Fig. 4d. Source data

**Extended Data Fig. 6:**
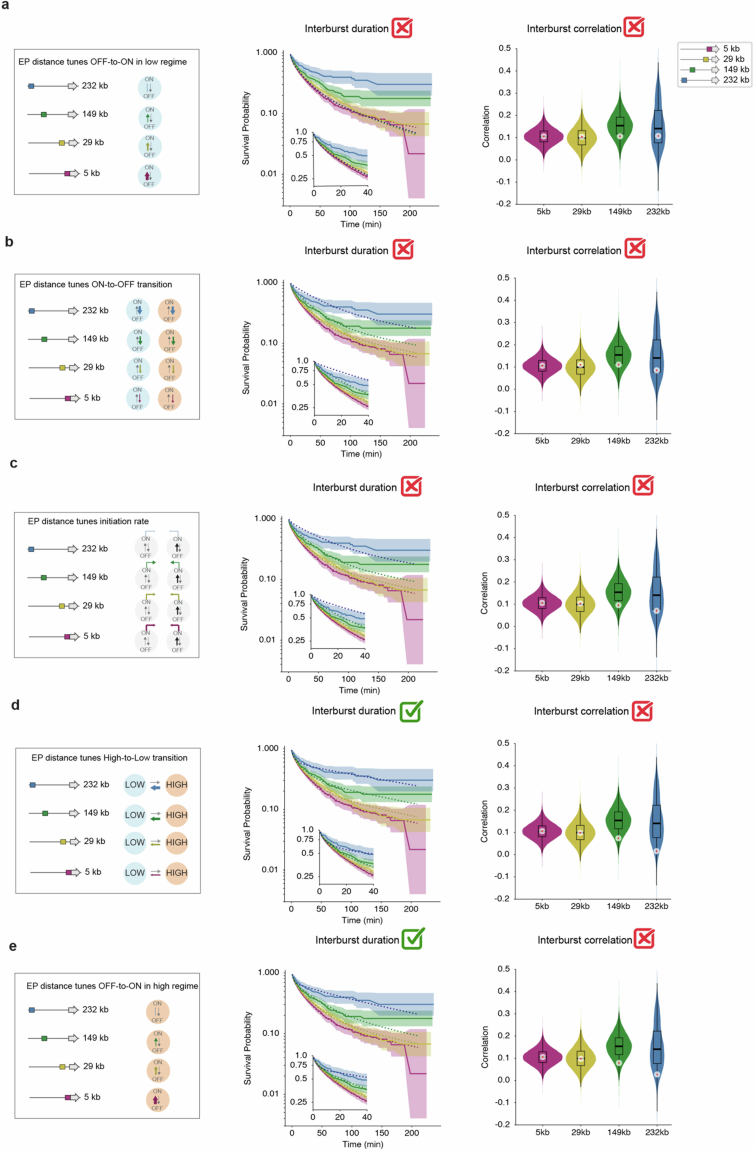
Best fit model. **a**–**e**, Best fit survival probabilities of the inter-burst duration of the 29 kb, 149 kb, and 232 kb cell lines (gold, green, and blue dotted lines) and predicted Pearson correlation of consecutive inter-burst periods (red crosses), for models where the enhancer-promoter genomic distance only affects the OFF-to-ON rate in the low-frequency regime (**a**), the ON-to-OFF rate (**b**), the initiation rate (**c**), the high-to-low rate (**d**), and the OFF-to-ON rate in the high-frequency regime (**e**). Data is like Fig. 6. Source data

We next tested whether data from the other clones could be explained under the stringent assumption that enhancer–promoter distance modulates only a single rate in the model. In principle, multiple rates could influence interburst durations while preserving the observed burst durations (Fig. 2c,d). Accordingly, for each rate, we fitted the model to the interburst survival distributions of the remaining clones (29 kb, 149 kb and 232 kb), allowing only that rate to vary while fixing all others to the best-fit values from the 5 kb clone. We then assessed whether the resulting parameters could reproduce the observed interburst correlations (Supplementary Note—Model analysis). The following three scenarios could not reproduce the interburst durations in the first place: (1) changing the On rate in the low-frequency regime, (2) changing the Off rate and (3) changing the initiation rate (Extended Data Fig. 6a–c). The following two other scenarios reproduced interburst durations well but failed to predict the trend and magnitude of interburst correlations: (1) changing the On rate in the high-frequency regime and (2) changing the high-to-low regime-switching rate (Extended Data Fig. 6d,e). Interestingly, only the scenario in which the low-to-high regime-switching rate depends on enhancer–promoter distance was able to fit the interburst distributions and simultaneously reproduce both the magnitude and trend of correlations between consecutive interburst intervals (Fig. 6a–c). Best agreement occurred with an average interburst duration of 1.0 h (enhancer–promoter distance = 29 kb), 1.6 h (distance = 149 kb) and 3.1 h (distance = 232 kb), resulting in burst frequencies of 1.4, 0.9 and 0.4 bursts per hour (Extended Data Fig. 5f). In this scenario, the low-to-high transition rate increased nonlinearly as a function of contact probability (Extended Data Fig. 5g), reflecting the nonlinear increase in burst frequency (Fig. 3a).

Taken together, our results are compatible with a model in which the promoter predominantly resides in a low-frequency bursting regime, where rare Off→On transitions generate the longest interburst timescale. Occasional transitions to a high-frequency regime produce intermediate interburst intervals (~25 min), while the shortest timescale (~3 min) reflects an initiation rate in the On state that is low relative to the rate of RNA release after transcription (Fig. 5b). This results in loss of fluorescence when no new RNA is initiated within the time required to elongate and release the previous transcript. Our analysis further indicates that many bursts correspond to single transcription events occurring sequentially within a single On state, forming clusters. When the promoter enters the high-frequency regime, such clusters occur more often. Notably, the enhancer controls the rate of switching to this regime, thereby regulating the effective burst frequency by modulating cluster occurrence. These processes operate out of thermodynamic equilibrium, as detailed balance is not satisfied.

**Extended Data Fig. 7:**
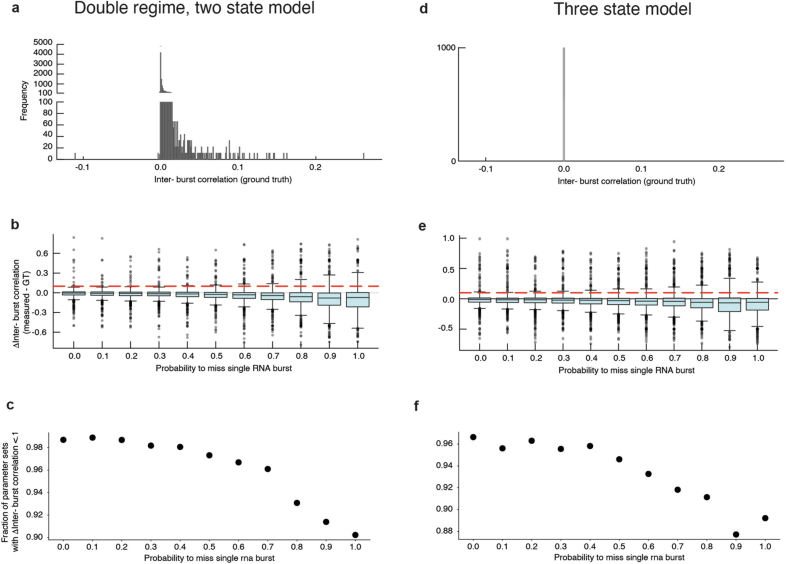
Robustness of inter-burst duration correlation to missed detection of weak bursts. Results are based on simulations of the double-regime, two-state model (**a**–**c**), and a three-state model with two sequential OFF states (**d**–**f**). For each model, 1000 parameter sets were used to generate synthetic burst trajectories. Panels **a** and **d** show the ground-truth inter-burst correlations corresponding to each parameter set. Panels **b** and **e** display the differences between the inferred and ground-truth correlations as a function of the probability of missing single-RNA bursts. Correlations were inferred from trajectories whose length distribution matches that of the experimental data. Boxes indicate the interquartile range (25th–75th percentiles), with the median shown as a horizontal line. Whiskers extend to the most extreme data points within 1.5× the interquartile range (IQR). Panels **c** and **f** summarize the results by showing the fraction of parameter sets for which differences presented in panels **b** and **e** are below the correlation measured in the data (0.1). Details of the procedure can be found in the Supplementary Note. Source data

**Extended Data Fig. 8:**
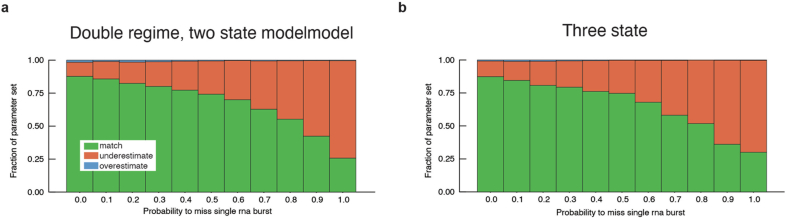
Robustness of inter-burst duration timescales to missed detection of weak bursts. Results are based on simulations of the double-regime, two-state model (**a**) and a three-state model with two sequential OFF states (**b**). For each model, 1,000 parameter sets were used to generate synthetic burst trajectories. Panels **a** and **b** display the fraction of parameter sets for which the inferred number of timescales matches (green), underestimates (orange), or overestimates (blue) the ground truth number of timescales, as a function of the probability of missing single-RNA bursts. The timescales were inferred from trajectories whose length distribution matches that of the experimental data. Details of the procedure can be found in the Supplementary Note—Model analysis. Source data

This model was motivated by the following two key observations: positive interburst correlations and the presence of three distinct interburst timescales. To exclude the possibility that these features arise from detection errors, particularly missed weak bursts, we analyzed simulated burst traces across thousands of parameter sets while systematically removing increasing fractions of weak events (Supplementary Note—Model analysis). These analyses indicate that it is highly unlikely that the missed detection of weak bursts leads to overestimation of the magnitude of correlations or the number of timescales (Extended Data Figs. 7 and 8).

Finally, the structure of the model we inferred from real-time transcriptional kinetics resembles a model we previously proposed to describe the static properties of the same experimental system^13^, although here we did not make any explicit assumptions about how regime transitions are linked to enhancer–promoter contacts (see ‘Discussion’).

## Discussion

Our study provides quantitative measurements of promoter burst kinetics as a function of genomic distance to an enhancer over a range representative of distances observed in mammalian genomes (from a few to hundreds of kb). By only modifying the position of the enhancer within a ‘neutral’ genomic region with minimal structural and regulatory complexity, we isolated the effect of genomic distance from other confounding factors such as enhancer–promoter biochemical compatibility and influences from additional regulatory sequences, which may confound interpretation of results when enhancer sequences are manipulated at endogenous gene loci.

In line with previous reports that enhancers may mainly affect burst frequency^11,19,20,22,30^, we observe that the genomic distance between the *Sox2* promoter and its SCR enhancer modulates the time between consecutive bursts, without any noticeable changes in burst size or duration (Fig. 2). We further reveal that bursts from an enhancer-driven promoter occur in clusters and that interburst durations are correlated and distributed across three well-separated timescales (Figs. 2 and 4 and Extended Data Fig. 3e).

The observed positive correlation between consecutive interburst durations is not compatible with models of promoter operation that consider a single-burst regime, and specifically two-state or three-state models of promoter operation^20,22,23^. Instead, we show that it can be generated by a simple alternative model in which the promoter switches between low-frequency and high-frequency two-state regimes that differ in their On rates. Testing this model against experimental perturbation of enhancer–promoter distance suggests that the enhancer controls the frequency at which clusters of bursts appear (Fig. 5), rather than the frequency at which single bursts occur within a cluster. We note that a key feature of this model is that transcription is initiated inefficiently from the On state, in line with recent interpretations of transcriptional kinetics in *Drosophila*^24^.

The structure of the model strongly resembles that of a model we previously proposed to describe population-based measurements in the same experimental system^13^. However, a key difference between the two models lies in the underlying assumptions about how regime transitions occur. In ref. ^13^, we hypothesized that switching from the low-frequency to high-frequency regime is triggered by stochastic enhancer–promoter interactions through one or more reversible regulatory steps, allowing entry into the high-frequency regime once completed. Under this assumption, the observed nonlinear relationship between contact probability and transcription forced the model to saturate at high contact probabilities, effectively implying that, at short genomic distances, the promoter operates exclusively in a high-frequency two-state regime. Here by moving beyond population-level measurements and directly quantifying promoter dynamics in real time, we find that this is not the case. Specifically, we observe three distinct interburst timescales, regardless of enhancer–promoter distance, indicating that promoter behavior cannot be fully captured by a simple two-state model, even at close genomic proximity. Although the simpler model we describe here does not explicitly consider enhancer–promoter interactions and their effect on the promoter, the finding that the low-to-high-regime transition rate depends on enhancer–promoter genomic distance (and thus on their contact probability; Extended Data Fig. 5g) still suggests that enhancer–promoter interactions determine regime switching. It also implies that the enhancer exerts a time-dependent effect on the promoter’s ‘On’ rate, possibly by temporarily increasing the availability of transcription factors or cofactors in the vicinity of the promoter.

We note that stochastic transitions from the low-frequency to the high-frequency regime were inferred to occur on average only once every 7, 10, 25 and 151 h when the enhancer is located at 5, 29, 149 and 232 kb, respectively (cf. low-to-high rates in Extended Data Fig. 5f). This suggests that encounters with the enhancer might only rarely translate into a detectable regulatory effect at the promoter, especially when the enhancer is located tens to hundreds of kb away. This is in line with our observation that a substantial number of cells do not burst over the course of 4 h (Extended Data Fig. 3f) and with other recent measurements on endogenous genes in mESC^20^.

The experiments reported here do not directly probe the dynamics of enhancer–promoter distances or how encounters translate into transcriptional events. Recent theoretical work and live imaging of nonregulatory DNA^31–33^ suggest that physical encounters (within a few tens of nm) between loci separated by ~150 kb occur every few tens of seconds. This is several 100-fold higher than the burst frequency measured here (approximately one burst every 3 h at 149 kb; Fig. 3a) and several 1,000-fold higher than the inferred transitions from the low-frequency to high-frequency regime (Extended Data Fig. 5e). If enhancer–promoter dynamics resemble those of nonregulatory DNA, these estimates imply that many consecutive encounters might be required to produce a single regime transition, likely through intermediate rate-limiting steps^13,34^. Alternatively, only a subset of encounters—for example, those exceeding a minimal duration^35^—may enable the transition from the low-frequency to high-frequency regime, which is then maintained by contact-independent processes, potentially involving chromatin modification, accessibility or Pol II recycling. This latter interpretation is supported by our recent finding that loop extrusion by cohesin results in rare but long-lived encounters between distal genomic sites^31^. Strikingly, the frequency and scaling of cohesin-driven long-lived encounters match those of the low-to-high regime transitions inferred here^31^. Moreover, because loop-extrusion-driven encounters require cohesin’s ATPase activity, they may help sustain the thermodynamic nonequilibrium cycle underlying the two-regime model.

In addition to controlling population-averaged and time-averaged transcription levels, enhancer position also modulates cell-to-cell variability in burst timing and number, with proximal enhancers producing more frequent and more uniform bursts than distal ones. This suggests that the large-scale organization of the *cis*-regulatory landscape strongly influences the accuracy and robustness of transcriptional programs^36^. It could therefore be expected that genes requiring rapid and reproducible transcriptional responses benefit from proximally located enhancers, whereas distal enhancers may be tolerated or favored when greater variability in timing or expression levels is permissible. Notably, a similar pattern has recently been observed in neurons^37,38^.

Finally, we note that bursts from the *Sox2* promoter in this experimental system are rarer than those previously detected at the endogenous *Sox2* locus^39,40^, even when the enhancer is in closer genomic proximity than the endogenous SCR. This might be due to the absence of the CTCF loops that connect the endogenous *Sox2* promoter and SCR^41^, the lack of additional enhancers^17,42^, or additional sequences that facilitate endogenous promoter operation^43^.

Taken together, our data uncover quantitative principles by which a distal enhancer controls transcription and its variability in single cells, and provide a quantitative framework for future studies to mechanistically address the interplay between enhancer–promoter interactions and transcriptional bursting.

## Methods

### Culture of mESCs

All cell lines are based on the E14Tg2a parental mESC line (karyotype: 19, XY; 129/Ola isogenic background). Cells were cultured on gelatin-coated culture plates in Glasgow Minimum Essential Medium (Sigma-Aldrich, G5154) supplemented with 15% fetal calf serum (Eurobio Abcys), 1% L-glutamine (Thermo Fisher Scientific, 25030024), 1% sodium pyruvate (Thermo Fisher Scientific, 11360039), 1% MEM nonessential amino acids (Thermo Fisher Scientific, 11140035), 100 μM β-mercaptoethanol (Thermo Fisher Scientific, 31350010) and 20 U ml^−1^ leukemia inhibitory factor (Miltenyi Biotec, premium grade) in 8% CO_2_ at 37 °C. For enhancer mobilization and subsequent experiments, the culture medium was additionally supplemented with 2i inhibitors (1 μM MEK inhibitor PD0325901 (Axon, 1408) and 3 μM GSK3 inhibitor CHIR 99021 (Axon, 1386)). Cells were tested regularly for mycoplasma contamination, and no contamination was detected.

### Targeting vectors for the integration of 24× MS2 stem-loops

The founder cell line carrying the piggyBac transgene and MS2 repeats is based on the founder cell line used in Fig. 1 of ref. ^13^. This cell line enables the piggyBac-mediated mobilization of the full-length SCR enhancer (lacking its internal CTCF site^13^) within a ‘neutral’ TAD on chromosome 15 from which two CTCF sites were deleted. The transgene was inserted at position chr15: 11,717,617 (mm10) in ref. ^13^.

We modified this cell line by inserting a 24× MS2 stem-loop cassette into the 3’ UTR of the split *EGFP* transcript. To knock-in the MS2 repeats^28^, we used three plasmids—a targeting vector, a gRNA vector to linearize the targeting vector and a gRNA vector targeting the 3’ UTR of the *EGFP* transcript at its genomic locus. The targeting vector has a pUC57 backbone (Genewiz) and contains 24× MS2 repeats flanked by homology arms and a bacterial gRNA sequence for plasmid linearization. To linearize the targeting vector, we used a modified pC2P plasmid including bacterial gRNA sequences and a Cas9-P2A-puromycin cassette, which was donated by D. Schübeler^44^. The gRNA sequence for knock-in of the MS2 cassette (targeting a position 3′ of the splitGFP gene and upstream of the SV40 polyA sequence) was designed using an online tool (https://eu.idtdna.com/site/order/designtool/index/CRISPR_SEQUENCE) and purchased from Microsynth AG. This gRNA sequence was cloned into the PX459 plasmid (Addgene, 48139) using a BsaI restriction site. Primers used are listed in Supplementary Table 3 and were ordered as desalted oligonucleotides from Microsynth.

### Generation of a founder cell line carrying 24× MS2 stem-loops

The founder line carrying the piggyBac transgene and MS2 repeats was generated by transfecting 1.5 × 10^6^ cells with 1.5 μg MS2 targeting vector, 750 ng PX459 (splitGFP_gRNA/Cas9) and 300 ng PX459 (linearization_gRNA/Cas9) using Lipofectamine 3000 (Thermo Fisher Scientific, L3000008) according to the manufacturer’s guidelines. Twenty-four hours after transfection, 1 µg ml^−1^ puromycin (InvivoGen, ant-pr-1) was added to the medium for 24 h. Cells were cultured in standard medium for an additional 4 days, after which single cells were isolated into 96-well plates. Sorted cells were kept for 2 days in medium supplemented with 100 μg ml^−1^ Primocin (InvivoGen, ant-pm-1) and 10 µM ROCK inhibitor (STEMCELL Technologies, Y-27632). Ten days after sorting, plates were duplicated and genomic DNA was extracted in situ by lysing cells with lysis buffer (100 mM Tris–HCl, pH 8.0, 5 mM EDTA, 0.2% SDS, 50 mM NaCl, 1 mg ml^−1^ proteinase K (Macherey-Nagel, 740506) and 0.05 mg ml^−1^ RNase (Thermo Fisher Scientific, EN0531)), followed by subsequent isopropanol precipitation. Individual cell lines were analyzed by genotyping PCR (genotyping primers listed in Supplementary Table 3) and correctly genotyped cell lines were expanded. Correct insertion was verified by Sanger sequencing of the genotyping PCR product, which covered the entire insert.

Stable integration of stdMCP-stdHaloTag was achieved via lentiviral transduction (gifted from J. Chao). Furthermore, 0.5 × 10^6^ cells were transduced using Polybrene Infection/Transfection Reagent (Sigma-Aldrich, TR-1003) according to the manufacturer’s guidelines, and 10 days later, cells were sorted for HaloTag expression into single cells as described above. After 10 days, one-third of the cells were replated onto 96-well glass-bottom microplates (Cellvis, P96-1.5H-N) and clonal lines were screened by microscopy for high SNR.

### Mobilization of the piggyBac enhancer cassette

Mobilization of the enhancer cassette was performed as previously described^13^ with minor adjustments. In brief, cells were cultured in medium additionally supplemented with 2i inhibitors. PiggyBac enhancer cassette mobilization was performed in 12 independent transfections by transfecting 3 × 10^5^ cells with 1 µg of an expression vector for the PBase transposase (gifted from J. Owens^45^). EGFP^+^ cell lines were then isolated using FACS into 96-well plates and expanded for DNA extraction, flow cytometry and freezing.

### Mapping piggyBac enhancer insertion sites in individual cell lines

PiggyBac integration sites were mapped using Splinkerette PCR after DNA extraction as described previously^13^. Splinkerette primers are listed in Supplementary Table 3. A modified script for analysis of the resulting Sanger sequencing reads is available on GitHub (https://github.com/fmi-basel/ggiorget_ms2analysis).

### Flow cytometry

EGFP levels of individual cell lines were measured in situ using a BD LSRII SORP flow cytometer equipped with a BD High Throughput Sampler. For each clone, measurements were performed in triplicate, and mean EGFP fluorescence intensities were calculated using FlowJo. All three replicates were averaged.

After expansion, before cell fixation and in parallel with live-cell imaging, EGFP levels of individual cell lines were measured in-tube using a BD LSRII SORP flow cytometer.

### Live-cell imaging

Next, 35-mm glass-bottom dishes (MatTek, P35G-1.5-14-C) were coated with 1–2 μg ml^−1^ laminin (Sigma-Aldrich, L2020) in PBS at 37 °C overnight. The day before imaging, 0.8–1 × 10^6^ cells were seeded in FluoroBrite DMEM (Gibco, A1896701) supplemented with culture medium containing 2i inhibitors (see Methods ‘Culture of mESCs’) and incubated at 37 °C, 8% CO_2_.

Right before imaging, Halo-tagged MCP was labeled with Halo-Ligand-Janelia Fluor 549 (Tocris Bioscience, custom synthesis based on 6503). To this end, the medium was replaced with fresh medium containing 100 nM Halo-Ligand for 20 min. Subsequently, cells were washed three times with PBS and fresh FluoroBrite medium was added.

Cells were imaged using a Nikon Eclipse Ti-E inverted widefield microscope equipped with a total internal reflection fluorescence (TIRF) microscopy iLAS2 module (Roper Scientific), a Perfect Focus System (Nikon), a motorized Z-piezo stage (ASI), Evolve 512 Delta EMCCD cameras with a pixel size of 16 × 16 μm^2^ (Photometrics) and a CFI APO TIRF ×100/1.49 numerical aperture (NA) oil-immersion objective (Nikon). The following laser lines were used: a 200-mW Toptica iBeam Smart laser at 488 nm and a 200-mW Coherent Sapphire laser at 561 nm. The microscope was operated in oblique illumination mode using VisiView software (Visitron). An enclosed microscope environmental control setup (The BOX and The CUBE, Life Science Instruments) was used at 37 °C and 8% CO_2_.

Before movie acquisition, one *z* stack (21 planes, spacing of 300 nm) of EGFP levels was recorded using 10% 488 nm laser power, an exposure time of 150 ms and a gain of 50. To image MS2 signals, a series of *z* stacks (21 planes, spacing of 300 nm) was acquired every 30 s using 4% 561 nm laser power, an exposure time of 100 ms and a gain of 100.

### Flatfield correction

Before movie acquisition, MatTek dishes filled with 20-nM Alexa Fluor 488 hydrazide (Thermo Fisher Scientific, A10436) or 100-nM Alexa Fluor 568 hydrazide (Thermo Fisher Scientific, A10437) were imaged using the same acquisition settings (see above). Two hundred *z* planes with 300 nm spacing were acquired and 21 planes close to the coverslip were subsequently selected. Three *z* stacks were averaged per day. Dark images were acquired by setting the laser power to 0%; 500 planes were acquired and averaged. Flatfield correction was performed using the following equation as a step of image processing (see below):

(Image – DarkImage) × mean(FlatFieldImage – DarkImage)/(FlatFieldImage – DarkImage)

### Image processing of live-cell movies

Raw images were *z* projected (mean projection for the 488-nm channel (EGFP), max projection for the 561-nm channel (MS2)) using Fiji and further processed using custom Python code. All code is available on GitHub at https://github.com/fmi-basel/ggiorget_ms2analysis.

Individual cells were segmented using StarDist and the ‘2D_versatile_fluo’ model^46,47^ and tracked over time using a linear assignment problem tracker with no gaps allowed, a minimal cell size and a minimal track length of 10 frames.

Active transcription spots were detected using Trackpy (after a band-pass filtering and thresholding; https://github.com/soft-matter/trackpy) and subsequently filtered by size and intensity to remove spurious detections.

Spots were linked over time within individual cells, and when no spot was visible between two bursts, the promoter position was linearly interpolated, accounting for lateral cell movement and relative deformation. If two spots were detected in the same cell at the same time, the brighter spot was chosen for linking.

Spot intensity was read out after projected images were flatfield-corrected (see above) using a circular mask (7-pixel diameter). The local background intensity was read out using a ring-shaped mask (1-pixel thickness) around the spot mask, separated by a 4-pixel gap. The global background intensity of the entire cell was read out by using the cell mask. SNR for individual spots was defined as the difference between spot intensity and local background intensity, divided by the standard deviation of the local background.

To construct intensity traces, spot and local background intensities were bleach-corrected by dividing by the normalized global background intensity. Subsequently, the local background intensity was subtracted from the spot intensity.

To segment transcriptionally active/inactive times, information on spot detection was used. Whenever a spot is detected, it is defined as a transcriptionally active time. To avoid confounding effects from declining fluorescence during the first hour of imaging (Extended Data Fig. 2a), the first hour of movies was excluded from analysis. To limit splitting interburst times by single-frame events, we removed 1-frame bursts from binarized traces and replaced them with zeroes.

Burst size was determined as the integrated intensity of the MS2 signal over an active transcription period. Burst amplitude was calculated as the maximum intensity during an active transcription period.

### Time-averaged probability to detect a burst in live-cell imaging

To calculate the time-averaged probability of observing a burst, binarized MS2 intensity traces (transcriptionally active/inactive times) were used. For each biological sample (cell line), the probability of observing a transcriptionally active time per time point was calculated and subsequently averaged over time.

### Survival probabilities calculated with the Kaplan–Meier estimator

To account for transcriptionally active/inactive times that start or end outside the imaging window (‘censored’ events), the Kaplan–Meier estimator^48^ was used to calculate survival probabilities of burst and interburst durations. This nonparametric method uses both uncensored (start and end within the imaging window) and right-censored data (start within the window). Tracks in which no burst was detected (censored on both sides) were excluded from the Kaplan–Meier calculation.

From binarized MS2 intensity traces, we calculated interburst and burst durations for both uncensored and right-censored events. The survival function *S*(*t*), representing the probability that an event exceeds time *t*, is expressed as:

$$S\left(t\right)=\prod _{{t}_{i}\le t}\left(1-\frac{{d}_{i}}{{n}_{i}}\right)$$

where ${t}_{i}$ represents a time length of at least one event (for example, one minute), *d*_*i*_ the number of events at time *t*_*i*_ (for example, number of active times at one minute), and *n*_*i*_ the number of events surviving (for example, number of active times longer than one minute, either uncensored or right-censored) up to *t*_*i*_.

The Python implementation ‘lifelines’ was used to compute the Kaplan–Meier estimator^49^.

### Survival probabilities of burst amplitude and burst size

Because burst amplitude or burst size is not lifetime data, the Kaplan–Meier estimator is not applicable for estimating their survival probabilities. Although censoring could, in principle, occur for these observables, in our dataset, most bursts were fully observed (2,924/2,973) due to their short duration. We therefore ignored censoring and computed empirical survival probabilities directly. Survival probabilities for burst size and burst amplitude were derived from MS2 intensity traces, and 95% confidence intervals for the resulting survival distributions were estimated by bootstrapping (*n* = 10,000).

### Mathematical model and parameter fitting

The double regime, two-state model was fitted to the data in the following two steps:

1. Single-distance fit—the model’s seven parameters were optimized using data from a single enhancer–promoter distance (5 kb). The model was fitted simultaneously to the survival probabilities of burst and interburst durations, the steady-state probability of observing a burst, and the correlation between consecutive interburst durations.
2. Multidistance fit—for other enhancer–promoter distances, only one parameter was fitted, while the remaining parameters were fixed at their 5-kb best-fit values. In this case, the model was fitted only to the survival probabilities of interburst durations.

The mathematical description and analysis of the model, the fitting procedures and the parameter sensitivity analysis are explained in detail in the Supplementary Note.

### Statistics and reproducibility

#### Live-cell imaging

Three to seven biological replicates were performed for each cell line, resulting in an average of 1,000–2,500 tracks per cell line. These tracks originate from approximately 200–400 cells per condition. We did not apply statistical methods to predetermine sample size and followed general standard practices in the field. These sample sizes ensure that burst statistics are robust to bootstrapping (Fig. 2). For flow cytometry measurements, three biological replicates per condition were performed.

### Reporting summary

Further information on research design is available in the Nature Portfolio Reporting Summary linked to this article.

## Online content

Any methods, additional references, Nature Portfolio reporting summaries, source data, extended data, supplementary information, acknowledgements, peer review information; details of author contributions and competing interests; and statements of data and code availability are available at 10.1038/s41588-026-02676-x.

## Supplementary information

Supplementary Information Supplementary Note (Model analysis and Gating strategy).

Reporting Summary

Peer Review file

Supplementary Table 1 Genomic coordinates (mm10) of SCR insertion sites.

Supplementary Table 2 Track and burst statistics.

Supplementary Table 3 List of primers.

Supplementary Video 1 Maximum projection (from a 10 µm *z* stack every 300 nm) of a movie of clone 5G7 (enhancer at −5 kb).

Supplementary Video 2 Maximum projection (from a 10 µm *z* stack every 300 nm) of a movie of clone 5F11 (enhancer at −149 kb).

## Source data

Source Data Figs. 1–6 and Extended Data Figs. 1–8 Source data.

## Data Availability

The raw and processed intensity trajectories from imaging data can be found on Zenodo (https://zenodo.org/records/17572944)^50^. Raw images are available from the corresponding author upon request. Source data are provided with this paper.

## Code availability

Image analysis code used in this study is available on GitHub (https://github.com/fmi-basel/ggiorget_ms2analysis) and archived on Zenodo (10.5281/zenodo.20280508)^51^. Code used for model inference, analysis and fitting is available on GitHub (https://github.com/gregroth/BurstingDynamicsInference) and archived on Zenodo (10.5281/zenodo.20266684)^52^.
