# Supplementary material for: Enhancer control of promoter activity and variability via frequency modulation of clustered transcriptional bursts
Source: Nat Genet. 2026 Jul 14;58(8):1967–75. doi: 10.1038/s41588-026-02676-x (PMC13447087; doi:10.1038/s41588-026-02676-x)
Supplement: Supplementary file 1 [file 41588_2026_2676_MOESM1_ESM.pdf]

nature genetics

Supplementary information https://doi.org/10.1038/s41588-026-02676-x

Enhancer control of promoter activity and variability via frequency modulation of clustered transcriptional bursts

In the format provided by the authors and unedited

Table of contents

Supplementary model analysis, p.1 to 12

Gating strategy, p. 13 to 16

Supplementary model analysis

In this Supplementary Information, we develop a theoretical framework to analyze the bursting dynamics of promoters with arbitrary combinatorial complexity. This framework provides analytical expressions for key observables, including the survival probabilities of burst and inter-burst durations, the steady-state probability of observing a burst, and the correlation between consecutive inter-burst periods. We then apply this framework to analyze and fit the double regime, two state model introduced in the main text.

The reminder of this document is structured as follows. Section 1 introduces the class of stochastic multi-state transcription models. Sections 2 derives analytical expressions for (1) the survival probabilities of the burst and inter-burst durations, (2) the steady-state probability of observing a burst, and (3) the correlation between consecutive inter-burst periods. Section 3 extends these expressions to account for the removal of singlet bursts from the data. Finally Sections 4 and 5 present the double regime, two state model and describe our method for fitting it to experimental data.

1 Generic stochastic multi-state model of transcription

We consider a generic multi-state transcription model where a promoter can occupy one of n states, denoted $\{s_1, \dots, s_n\}$, and undergo stochastic transitions between them at rates k_{ij}. From state s_i, transcription is initiated stochastically at rate μ_i. States with $\mu_i = 0$ are "OFF" states, and states with $\mu_i > 0$ are "ON" states. Upon initiation, an mRNA stem loop reporter forms instantaneously and is stochastically released from DNA at a rate δ. The kinetic reactions governing this model are:

$$
\begin{aligned}
s_i \quad &\underset{k_{ji}}{\overset{k_{ij}}{\rightleftharpoons}} \quad s_j, \ \ \forall i \neq j, \ \ i,j \in \{1,\dots,n\} \\
s_i \quad &\underset{\mu_i}{\rightarrow} \quad s_i + \text{mRNA}, \quad \forall i \in \{1,\dots,n\} \\
\text{mRNA} \quad &\underset{\delta}{\rightarrow} \quad \emptyset
\end{aligned}
$$

This system defines a continuous-time Markov chain, $Z_t = (z_1(t), z_2(t))$, where $z_1(t)$ represents the promoter state at time t, and $z_2(t)$ represents the number of nascent mRNA at time t.

1.1 Linear indexing of the state space

To facilitate computations, we introduce a *linear indexing function* mapping the pair (z_1, z_2) to a single "hyper" state $\phi(z_1, z_2) \in \mathbb{N}$, defined as:

$$\phi(i, m) = n(m-1) + i, \tag{1}$$

with its inverse

$$\phi^{-1}(k) = (k \bmod n, \lfloor \frac{k}{n} \rfloor). \tag{2}$$

In other words, this indexing orders the state space such that the first n states correspond to promoter states with 0 mRNA, the next n states correspond to promoter states with 1 mRNA, and so forth. The following scheme illustrates the linear indexing for 5 promoter states:

$$\underbrace{s_1 \quad s_2 \quad s_3 \quad s_4 \quad s_5}_{\text{mRNA = 0}} \quad \underbrace{s_1 \quad s_2 \quad s_3 \quad s_4 \quad s_5}_{\text{mRNA = 1}} \quad \underbrace{s_1 \quad s_2 \quad s_3 \quad s_4 \quad s_5}_{\text{mRNA = 2}} \quad \cdots$$

1.2 Infinitesimal generator of the process Z_t

The infinitesimal generator of the continuous-time Markov chain Z_t is the matrix $\mathbf{Q}$ containing all transition rates, with each row summing to zero. Based on the definition of the multi-state transcription model, the generator decomposes as

$$\mathbf{Q} := \mathbf{Q}_p + \mathbf{T} + \mathbf{D} \tag{3}$$

where:

- $\mathbf{Q}_p$ governs promoter state transitions for each number of nascent mRNA,
- $\mathbf{T}$ describes transcription initiation,
- $\mathbf{D}$ accounts for mRNA release.

The matrix $\mathbf{Q}_p$ describes transitions between promoter states while maintaining the same number of nascent mRNA:

$$\mathbf{Q}_p = \mathbf{I} \otimes \mathbf{K} \tag{4}$$

where $\mathbf{I}$ is the identity matrix, $\mathbf{K} = (k_{ij})$ is the $n \times n$ promoter transition matrix, and $\otimes$ is the Kronecker product.

The matrix $\mathbf{T}$ describes the transition from m to $m+1$ mRNA:

$$\mathbf{t}_{i,j} = \begin{cases} \mu_{\{i \bmod n\}}, & \text{if } j = i + n \\ 0, & \text{otherwise} \end{cases} \tag{5}$$

The matrix $\mathbf{D}$ describes the transition from m to $m-1$ mRNA:

$$\mathbf{d}_{i,j} = \begin{cases} \lfloor i/n \rfloor \delta, & \text{if } j = i - m \\ 0, & \text{otherwise} \end{cases} \tag{6}$$

1.3 Finite-state approximation

The state space of Z_t is infinite because the number of nascent mRNA is unbounded. However, in practice, this number remains small. Inspired by the finite state projection method (Munsky and Khammash [2006]), we approximate the system by truncating the state space. Effectively, we define a maximal number of nascent mRNA per cell, M_{max}, leading to a finite state space:

$$\tilde{Z}_t \in \{1, \dots, n\} \times \{0, \dots, M_{\text{max}}\}. \tag{7}$$

The infinitesimal generator of the truncated process, $\tilde{\mathbf{Q}}$, has entries:

$$\tilde{q}_{i,j} = \begin{cases} q_{ij}, & \text{if } i \neq j \\ -\sum_{k \leq n(M_{\text{max}}+1), k \neq i} q_{i,k} & \text{if } i = j \end{cases} \tag{8}$$

1.4 Discretization of the generic model

Experimental observations are acquired every 30 seconds. To match this time resolution, we define the discretized process:

$$X_k := \tilde{Z}_{k\Delta t}, \tag{9}$$

where $\Delta t = 30s$. The process X_k forms a discrete-time Markov chain in $\{1, \dots, n\} \times \{0, \dots, M_{\text{max}}\}$, with transition matrix $\mathbf{P} = (p_{i,j})$, computed directly from the infinitesimal generator $\tilde{\mathbf{Q}}$ via:

$$p_{ij} = \mathbb{P}(X_{k+1} = j | X_k = i) = (e^{\Delta t \tilde{\mathbf{Q}}})_{ij}. \tag{10}$$

The steady-state distribution $\boldsymbol{\pi}$ of the Markov chain X is the left eigenvector of the transition matrix $\mathbf{P}$ associated with the eigenvalue 1.

1.5 What is a *burst* in the model?

In our experiments, the final readout is a binary track for each cell, where 1 and 0 correspond to the presence or absence of nascent mRNA at the gene, respectively. A ”burst” is defined as a period of consecutive ones. In the model, we define a similar binary process B_t, where

- $B_t = 1$ if there is at least one nascent mRNA at the promoter,
- $B_t = 0$ ohtherwise

Using the linear indexing of the state space, we define the set of non-bursting states:

$$\mathcal{N} = \{1, \dots, n\}, \tag{11}$$

and the set of ”bursting” states:

$$\mathcal{B} = \{n+1, \dots, n(M_{max}+1)\}. \tag{12}$$

Thus, the burst process is given by:

$$B_t = \begin{cases} 1, & \text{if } X_t \in \mathcal{B} \\ 0 & \text{if } X_t \in \mathcal{N}. \end{cases} \tag{13}$$

2 Computation of the observables in the generic model

In this section we derive matrix-based formulas to compute the survival probabilities of burst and inter-burst durations, the steady-state probability of observing a burst, and the correlation between consecutive inter-burst durations. These calculations ultimately reduce to computing the eigenvector of $\mathbf{P}$ corresponding to eigenvalue 1 (steady-state distribution), inverting matrices of the form $\mathbf{I} - \mathbf{P}_{\mathcal{A}}$ for sub-matrices $\mathbf{P}_{\mathcal{A}}$ of the matrix $\mathbf{P}$, and performing direct matrix multiplications.

2.1 Survival probabilities of the burst durations

To compute the distribution of burst durations, we define a new absorbing Markov chain $X^{\mathcal{B}}$, which modifies the original process X by making all non-bursting states ($\mathcal{N}$) absorbing. In other words, the modified process $X^{\mathcal{B}}$ evolves like the original process X until it reaches a state with zero nascent mRNA, at which point it stops permanently. The transition matrix of the absorbing Markov chain $X^{\mathcal{B}}$ is:

$$\tilde{\mathbf{P}} = \begin{bmatrix} \mathbf{P}_{\mathcal{BB}} & \mathbf{P}_{\mathcal{BN}} \\ \mathbf{1} & \mathbf{0} \end{bmatrix} \tag{14}$$

where $\mathbf{P}_{\mathcal{AB}}$ represents the transitions between states in $\mathcal{A}$ and $\mathcal{B}$.

The probability that a burst, starting from state i in the original process X, lasts longer than time t is equivalent to the probability that a burst, starting from i in the modified process $X^{\mathcal{B}}$, has not yet been absorbed at time t:

$$\mathbb{P}(T_{\text{Burst}} > t | X_0^{\mathcal{B}} = i) = \sum_{j \in \mathcal{B}} (\mathbf{P}^t_{\mathcal{BB}})_{ij}. \tag{15}$$

To compute the steady-state probability of a burst lasting more than t, we need to determine the steady-state distribution of states at which bursts start, denoted by $\mathbf{p}^{\mathcal{B}}$:

$$\begin{aligned} p_j^{\mathcal{B}} &:= \mathbb{P}(\text{Burst starts in state } j) \\ &= \mathbb{P}(X_{t+1} = j | X_t \in \mathcal{N}, X_{t+1} \in \mathcal{B}) \\ &= \frac{\mathbb{P}(X_{t+1} = j, X_t \in \mathcal{N})}{\mathbb{P}(X_{t+1} \in \mathcal{B}, X_t \in \mathcal{N})} \end{aligned} \tag{16}$$

We expend the numerator using the Markov property:

$$\begin{aligned} \mathbb{P}(X_{t+1} = j, X_t \in \mathcal{N}) &= \sum_{i \in \mathcal{N}} \mathbb{P}(X_{t+1} = j, X_t = i) \\ &= \sum_{i \in \mathcal{N}} \mathbb{P}(X_{t+1} = j | X_t = i) \mathbb{P}(X_t = i) \end{aligned}$$

Expressed in terms of the steady-state probability distribution $\boldsymbol{\pi}$ of X and its transition matrix $\mathbf{P}$,

$$\mathbb{P}(X_{t+1} = j, X_t \in \mathcal{N}) = \sum_{i \in \mathcal{N}} p_{ij} \pi_i \tag{17}$$

Substituting equation (17) in equation (16), we obtain

$$\mathbf{p}^{\mathcal{B}} = \frac{1}{\boldsymbol{\pi}^{\mathcal{N}} \mathbf{P}_{\mathcal{NB}} \mathbf{e}} \boldsymbol{\pi}^{\mathcal{N}} \mathbf{P}_{\mathcal{NB}}$$

where $\boldsymbol{\pi}^{\mathcal{N}}$ is the row vector with entries describing the steady-state probabilities of the non-bursting states, and $\mathbf{e}$ is the column vector with all entries equal to 1.

Combining the previous results, the probability that a burst lasts for more than time t is given by:

$$\mathbb{P}(T_{\text{Burst}} > t) = \mathbf{p}^{\mathcal{B}} \mathbf{P}^t_{\mathcal{B}\mathcal{B}} \mathbf{e}. \tag{18}$$

2.2 Survival probabilities of the inter-burst durations

The survival probability of inter-burst durations is computed using the same strategy as for the burst durations, but with the bursting states $\mathcal{B}$ as absorbing. First, we define the steady-state distribution of the states where an inter-burst period begins:

$$\mathbf{p}^{\mathcal{N}} = \frac{1}{\boldsymbol{\pi}^{\mathcal{B}} \mathbf{P}_{\mathcal{B}\mathcal{N}} \mathbf{e}} \boldsymbol{\pi}^{\mathcal{B}} \mathbf{P}_{\mathcal{B}\mathcal{N}} \tag{19}$$

where $\boldsymbol{\pi}^{\mathcal{B}}$ is the row vector with entries describing the steady-state probabilities of the bursting states, and $\mathbf{e}$ is the column vector with all entries equal to 1. The survival probability of an inter-burst durations is then:

$$\mathbb{P}(T_{\text{Inter-burst}} > t) = \mathbf{p}^{\mathcal{N}} \mathbf{P}^t_{\mathcal{N}\mathcal{N}} \mathbf{e}. \tag{20}$$

2.3 Characteristic timescales of the inter-burst periods

Using the spectral decomposition of the transition matrix $\mathbf{P}_{\mathcal{N}\mathcal{N}}$, we can express the survival probability function of the inter-burst periods as a sum of exponential terms. Let

$$\mathbf{P}_{\mathcal{N}\mathcal{N}} = V \mathbf{D} V^{-1}$$

denote the eigendecomposition of $\mathbf{P}_{\mathcal{N}\mathcal{N}}$, where $V = [v_1, \ldots, v_n]$ is the matrix of right eigenvectors, $V^{-1} = [u_1, \ldots, u_n]^\top$ is the matrix whose rows are the corresponding left eigenvectors, and $\mathbf{D} = \operatorname{diag}(\lambda_1, \ldots, \lambda_n)$ contains the eigenvalues. Then, the survival probability of the inter-burst duration can be written as

$$\mathbb{P}(T_{\text{inter-burst}} > t) = \sum_{i \in \mathcal{N}} (\mathbf{p}^{\mathcal{N}} v_i)\,(u_i^\top \mathbf{e})\, e^{t \log(\lambda_i)}.$$

The characteristic timescales of the inter-burst periods are given by $\{\log(\lambda_i)\}$, and their weights by $\{(\mathbf{p}^{\mathcal{N}} v_i)(u_i^\top \mathbf{e})\}$.

2.4 Probability to observe a burst

The steady state probability to observe a burst is given by the probability that the Markov chain X is in a bursting state at steady-state:

$$\begin{aligned}\mathbb{P}(\text{to observe a burst}) &= \mathbb{P}(X_t \in \mathcal{B}) \\ &= \sum_{i \in \mathcal{B}} \pi_i\end{aligned}$$

2.5 Correlation between consecutive inter-burst periods

Let T_1 and T_2 denote the durations of two consecutive inter-burst periods. Their correlation is defined as:

$$\rho_{T_1,T_2} := \frac{\mathrm{Cov}(T_1, T_2)}{\sigma_{T_1}\sigma_{T_2}} \tag{21}$$

where $\mathrm{Cov}(T_1, T_2) = \mathbb{E}[(T_1 - \mathbb{E}[T_1])(T_2 - \mathbb{E}[T_2])]$ is the covariance between the two durations, and $\sigma_T = \sqrt{\mathrm{Var}(T)}$ is the standard deviation of T. Since T_1 and T_2 are identically distributed, we can express the covariance as:

$$\mathrm{Cov}(T_1, T_2) = \mathbb{E}[T_1 T_2] - \mathbb{E}[T_1]^2, \tag{22}$$

and the denominator simplifies to:

$$\sigma_{T_1}\sigma_{T_2} = \mathrm{Var}(T_1) = \mathbb{E}[T_1^2] - \mathbb{E}[T_1]^2.$$

To compute the expectations $\mathbb{E}[T_1^2]$, $\mathbb{E}[T_1]$, $\mathbb{E}[T_1 T_2]$, we use the absorbing Markov chains introduced in Sections 2.1 and 2.2:

- $X^{\mathcal{N}}$, which evolves like the original process X until it reaches a bursting state, where it is absorbed.
- $X^{\mathcal{B}}$, which evolves like X until it reaches a non-bursting state, where it is absorbed.

The inter-burst duration T_1 is equivalent to the time before absorption in the Markov chain $X^{\mathcal{N}}$. A key element for computing absorbing times is the fundamental matrix,

$$\mathbf{N}^{\mathcal{N}} = (\mathbf{I} - \mathbf{P}_{\mathcal{N}\mathcal{N}})^{-1}. \tag{23}$$

The $(i-j)$-th entry of $\mathbf{N}^{\mathcal{N}}$ represents the expected time spent in state j given that $X^{\mathcal{N}}$ started in state i. Similarly, the fundamental matrix for $X^{\mathcal{B}}$ is:

$$\mathbf{N}^{\mathcal{B}} = (\mathbf{I} - \mathbf{P}_{\mathcal{B}\mathcal{B}})^{-1}. \tag{24}$$

Following Iosifescu [1980], the first and second moments of the absorbing time are:

$$\begin{aligned} \mathbb{E}[T_i] &= \mathbf{p}^{\mathcal{N}}\mathbf{N}^{\mathcal{N}}\mathbf{1} && (25)\\ \mathbb{E}[T_i^2] &= \mathbf{p}^{\mathcal{N}}\left((2\mathbf{N}^{\mathcal{N}} - \mathbf{I})\mathbf{N}^{\mathcal{N}}\mathbf{1}\right) && (26)\\ & && (27) \end{aligned}$$

for $i = 1, 2$, where $\mathbf{p}^{N}$ is the steady-state probability of the initial state of an inter-burst event, as defined in equation (19).

Unlike $\mathbb{E}[T_1]$ and $\mathbb{E}[T_1^2]$, the expectation $\mathbb{E}[T_1 T_2]$ cannot be directly obtained from the absorbing Markov chain $X^{\mathcal{N}}$, since it depends on the burst separating the two inter-burst periods. Using conditional probability, we expand:

$$E[T_1 T_2] = \sum_{t \geq 0} t\mathbb{E}[T_2 | T_1 = t]\mathbb{P}(T_1 = t). \tag{28}$$

By conditioning on the final state of the first inter-burst period and using the conditional lay of total expectation:

$$\mathbb{E}[T_2 \mid T_1 = t] = \sum_{j \in \mathcal{B}} \mathbb{E}[T_2 \mid X_{T_1} = j]\,\mathbb{P}(X_{T_1} = j \mid T_1 = t). \tag{29}$$

This equality holds because T_2 is conditionally independent of T_1 given X_{T_1} as a consequence of the Markov property. Further conditioning on the state at the end of the burst separating T_1 and T_2:

$$\mathbb{E}[T_2 \mid X_{T_1} = j] = \sum_{k\in\mathcal{N}} \mathbb{E}[T_2 \mid X_\tau = k]\, \mathbb{P}(X_\tau = k \mid X_{T_1} = j), \tag{30}$$

where τ is the burst duration separating the two inter-burst events. Substituting, we obtain:

$$\mathbb{E}[T_1T_2] = \sum_{t\geq 0}\sum_{j\in\mathcal{B}}\sum_{k\in\mathcal{N}} t\mathbb{E}[T_2 \mid X_\tau = k]\, \mathbb{P}(X_\tau = k \mid X_{T_1} = j)\mathbb{P}(X_{T_1} = j \mid T_1 = t)\mathbb{P}(T_1 = t). \tag{31}$$

The second term in equation (31) is the expectation of the inter-burst duration, given the initial state k, which correspond to the time to absorption of the Markov chain $X^{\mathcal{N}}$ given its initial state:

$$\mathbb{E}[T_2|X_\tau = j] = (\mathbf{N}^{\mathcal{N}}\mathbf{e})_j. \tag{32}$$

The third term in equation (31) is the probability that the Markov chain $X^{\mathcal{B}}$ is absorbed in the non-bursting state k, given that it starts in the bursting state j. Following Iosifescu [1980], this probability corresponds to the j,k entry of the matrix $\mathbf{N}^{\mathcal{B}}\mathbf{P}_{\mathcal{B}\mathcal{N}}$:

$$\mathbb{P}(X_\tau = k|X_{T_1} = j) = (\mathbf{N}^{\mathcal{B}}\mathbf{P}_{\mathcal{B}\mathcal{N}})_{jk}. \tag{33}$$

The fourth term in equation (31) is the distribution of states at the end of the first inter-burst duration, conditioned on the duration of the inter-burst:

$$\begin{aligned}
\mathbb{P}(X_{T_1} = j|T_1 = t) &= \mathbb{P}(X_t = j|T_1 = t) \\
&= \frac{\mathbb{P}(X_t = j, T_1 = t)}{\mathbb{P}(T_1 = t)} \\
&= \frac{1}{\mathbb{P}(T_1 = t)} \sum_{\ell\in\mathcal{N}} \mathbb{P}(X_t^{\mathcal{N}} = j, X_{t-1}^{\mathcal{N}} = \ell) \\
&= \frac{1}{\mathbb{P}(T_1 = t)} \sum_{\ell\in\mathcal{N}} \mathbb{P}(X_t^{\mathcal{N}} = j|X_{t-1}^{\mathcal{N}} = \ell)\mathbb{P}(X_{t-1}^{\mathcal{N}} = \ell) \\
&= \frac{1}{\mathbb{P}(T_1 = t)} \sum_{\ell\in\mathcal{N}}\sum_{q\in\mathcal{N}} \mathbb{P}(X_t^{\mathcal{N}} = j|X_{t-1}^{\mathcal{N}} = \ell)\mathbb{P}(X_{t-1}^{\mathcal{N}} = \ell|X_0^{\mathcal{N}} = q)\mathbb{P}(X_0^{\mathcal{N}} = q) \\
&= \frac{1}{\mathbb{P}(T_1 = t)} \sum_{\ell,q\in\mathcal{N}} (\mathbf{P}_{\mathcal{N}\mathcal{B}})_{\ell j}(\mathbf{P}_{\mathcal{N}\mathcal{N}}^{t-1})_{q\ell}\mathbf{p}_q^{\mathcal{N}}
\end{aligned} \tag{34}$$

Substituting equations (32), (33), and (34) into equation (31), we obtain the final matrix formulation:

$$E[T_1T_2] = \sum_{t\geq 0} t\mathbf{p}^{\mathcal{N}}\mathbf{P}_{\mathcal{N}\mathcal{N}}^{t-1}\mathbf{P}_{\mathcal{N}\mathcal{B}}N_{\mathcal{B}}\mathbf{P}_{\mathcal{B}\mathcal{N}}\mathbf{N}_{\mathcal{N}}\mathbf{e} \tag{35}$$

3 Computation of the observables after removing singlet bursts

In the final bursting track obtained for each cell, singlet bursts (i.e., bursts lasting only one frame) were filtered out. To ensure a consistent comparison between the experimental data and the model, we must apply the same filtering to the model-generated bursts. For burst durations, this is straightforward: we simply condition on bursts lasting more than one frame.

For inter-burst durations, filtering is more complex because removing a singlet burst merges two consecutive inter-burst periods, altering their distribution.

To systematically remove singlet bursts in the process X, we expand the state space by introducing a set of *pre-bursting* states, $\mathcal{B}^p$, which are copies of the bursting states. The new state space consists of:

$$\begin{aligned}\mathcal{N} &= \{1,\ldots,n\}\\ \mathcal{B}^p &= \{n+1,\ldots,n(M_{max}+1)\}\\ \mathcal{B} &= \{n(M_{max}+1)+1,\ldots,1n(M_{max}+1\}.\end{aligned}$$

The key idea is to redirect the original process X into $\mathcal{B}^p$ whenever it enters a bursting state, and then return it to the original state space in the next time step. In this way, we can discard the single visit in the pre-bursting states which correspond to the singlet bursts of the original process. Formerly, we define a modified process, Y_t, which evolves like X_t, except when entering a bursting state. Specifically, if X_t enters the bursting state i at time t (i.e. $X_t = i$), the modified process Y_t transitions into the corresponding pre-bursting state in $\mathcal{B}^p$ (i.e. $Y_t = i + nM_{max}$). In the next time step, the process Y goes back to the original state space by following the same transition as X, i.e., $Y_{t+1} = X_{t+1}$.

We define the new binary process, $\tilde{B}_t$, that excludes singlet bursts as:

$$\tilde{B}_t = \begin{cases} 1, & \text{if } Y_t \in \mathcal{B} \\ 0 & \text{if } Y_t \in \mathcal{N} \cup \mathcal{B}^p \end{cases} \tag{36}$$

The trajectories of $\tilde{B}_t$ match those of B_t after singlet bursts are removed, except that bursts in $\tilde{B}_t$ are one time unit shorter because time spent in $\mathcal{B}^p$ is unrecorded. Thus, the probability that the inter-burst duration in B_t (with singlet bursts removed) exceeds t is equivalent to the probability that the inter-burst duration in $\tilde{B}_t$ exceeds $t+1$:

$$\mathbb{P}(T_{\text{Inter-burst, w/o singlets}} > t) = \mathbb{P}(\tilde{T}_{\text{Inter-burst}} > t+1) \tag{37}$$

The transition matrix $\tilde{\mathbf{P}}$ of the process Y is structured as:

$$\tilde{\mathbf{P}} = \begin{array}{c} \\ \end{array}\begin{array}{c}\begin{array}{ccc} \mathcal{N} & \mathcal{B}^p & \mathcal{B} \end{array}\\ \left[\begin{array}{c|c|c} \mathbf{P}_{\mathcal{N}\mathcal{N}} & \mathbf{P}_{\mathcal{N}\mathcal{B}} & \mathbf{0} \\ \hline \mathbf{P}_{\mathcal{B}\mathcal{N}} & \mathbf{0} & \mathbf{P}_{\mathcal{B}\mathcal{B}} \\ \hline P_{\mathcal{B}\mathcal{N}} & \mathbf{0} & \mathbf{P}_{\mathcal{B}\mathcal{B}} \end{array}\right]\end{array} \begin{array}{c} \\ \mathcal{N} \\ \mathcal{B}^p \\ \mathcal{B} \end{array} \tag{38}$$

To simplify notation, wemerge the original non bursting states $\mathcal{N}$ and pre-bursting states $\mathcal{B}^p$ into a single set $\tilde{\mathcal{N}}$. The transition matrix then simplifies to :

$$\tilde{\mathbf{P}} = \begin{bmatrix} \mathbf{P}_{\tilde{\mathcal{N}}\tilde{\mathcal{N}}} & \mathbf{P}_{\tilde{\mathcal{N}}\mathcal{B}} \\ \mathbf{P}_{\mathcal{B}\tilde{\mathcal{N}}} & \mathbf{P}_{\mathcal{B}\mathcal{B}} \end{bmatrix} \tag{39}$$

3.1 Survival probabilities of the inter-burst durations

To compute the survival probability of the inter-burst duration after removing the singlet bursts, we apply the strategy outlined in Section 2.1 to the Markov chain Y, treating bursting states ($\mathcal{B}$) as absorbing. From equation (37), we obtain

$$\mathbb{P}(T_{\text{Inter-burst, wo singlets}} > t) = \mathbf{p}^{\tilde{\mathcal{N}}}\mathbf{P}_{\tilde{\mathcal{N}}\tilde{\mathcal{N}}}^{t+1}\mathbf{e}, \tag{40}$$

where $\mathbf{p}^{\tilde{\mathcal{N}}}$ is the steady-state distribution of states where an inter-burst period starts in the Markov chain Y.

3.2 Characteristic timescales of the inter-burst periods

The characteristic timescales and their weights can be computed using the same reasoning as in Section 2.3. The characteristic timescales of the inter-burst periods are therefore given by $\{\log(\lambda_i)\}$, and their corresponding weights by $\{(\mathbf{p}^{\tilde{\mathcal{N}}}\mathbf{P}_{\tilde{\mathcal{N}}\tilde{\mathcal{N}}}v_i)(u_i^\top \mathbf{e})\}$, where $\{\lambda_i\}$ are the eigenvalues of the matrix $\mathbf{P}_{\tilde{\mathcal{N}}\tilde{\mathcal{N}}}$, and $\{v_i\}$ and $\{u_i\}$ denote its right and left eigenvectors, respectively.

3.3 Correlation between consecutive inter-burst durations

To compute the correlation between consecutive inter-burst durations after removing the singlet bursts, we apply the strategy outlined in Section 2.5 to the Markov chain Y. Let

- T_1^w and T_2^w be the durations of two consecutive inter-burst periods in the original Markov chain X after singlet bursts are removed.
- $\tilde{T}_1$ and $\tilde{T}_2$ be the corresponding durations in the Markov chain Y.

Since $T_i^w = \tilde{T}_i + 1$, the correlation between T_1^w and T_2^w is equivalent to the one between $\tilde{T}_1$ and $\tilde{T}_2$, leading to:

$$\rho_{T_1^w,T_2^w} = \frac{\mathbb{E}[\tilde{T}_1\tilde{T}_2] - \mathbb{E}[\tilde{T}_1]^2}{\mathbb{E}[\tilde{T}_1^2] - \mathbb{E}[\tilde{T}_1]^2}. \tag{41}$$

Following equations (26), (27) and (35), we compute each term in equation (41) as follows:

$$\mathbb{E}[T_i^w] = \mathbf{p}^{\tilde{\mathcal{N}}}\tilde{\mathbf{N}}\mathbf{1} \tag{42}$$

$$\mathbb{E}[(T_i^w)^2] = \mathbf{p}^{\tilde{\mathcal{N}}}\left((2\tilde{\mathbf{N}}^{\tilde{\mathcal{N}}} - \mathbf{I})\tilde{\mathbf{N}}^{\tilde{\mathcal{N}}}\mathbf{1}\right) \tag{43}$$

$$E[T_1^w T_2^w] = \sum_{t\geq 0} t\mathbf{p}^{\tilde{\mathcal{N}}}\mathbf{P}_{\tilde{\mathcal{N}}\tilde{\mathcal{N}}}^{t-1}\mathbf{P}_{\tilde{\mathcal{N}}\mathcal{B}}\mathbf{N}_{\mathcal{B}}\mathbf{P}_{\mathcal{B}\tilde{\mathcal{N}}}\tilde{\mathbf{N}}_{\tilde{\mathcal{N}}}\mathbf{e} \tag{44}$$

4 Double regime, two state model

The double regime, two state model is a stochastic, multi-state transcription model where the promoter can switch between two different transcriptional regimes: 'low-frequency' and 'high-frequency' regimes. In each regime, the promoter has an ON state, where transcription initiates at rate μ, and an OFF state, where transcription is inactive. The only difference between the two regimes is the OFF-to-ON transition rates: $k_o n^L$ in the low-frequency regime and k_{on}^H in the high-frequency, where $k_{on}^L < k_{on}^H$. In both regimes, the ON-to-OFF transition rate is k_{off}, and each RNA is released from DNA at rate δ. The promoter can switch between regimes at rates k_f from low- to high-frequency regime, and k_b from high- to the low-frequency regime. This model consists of four states (two ON and two OFF), with the following kinetic scheme:

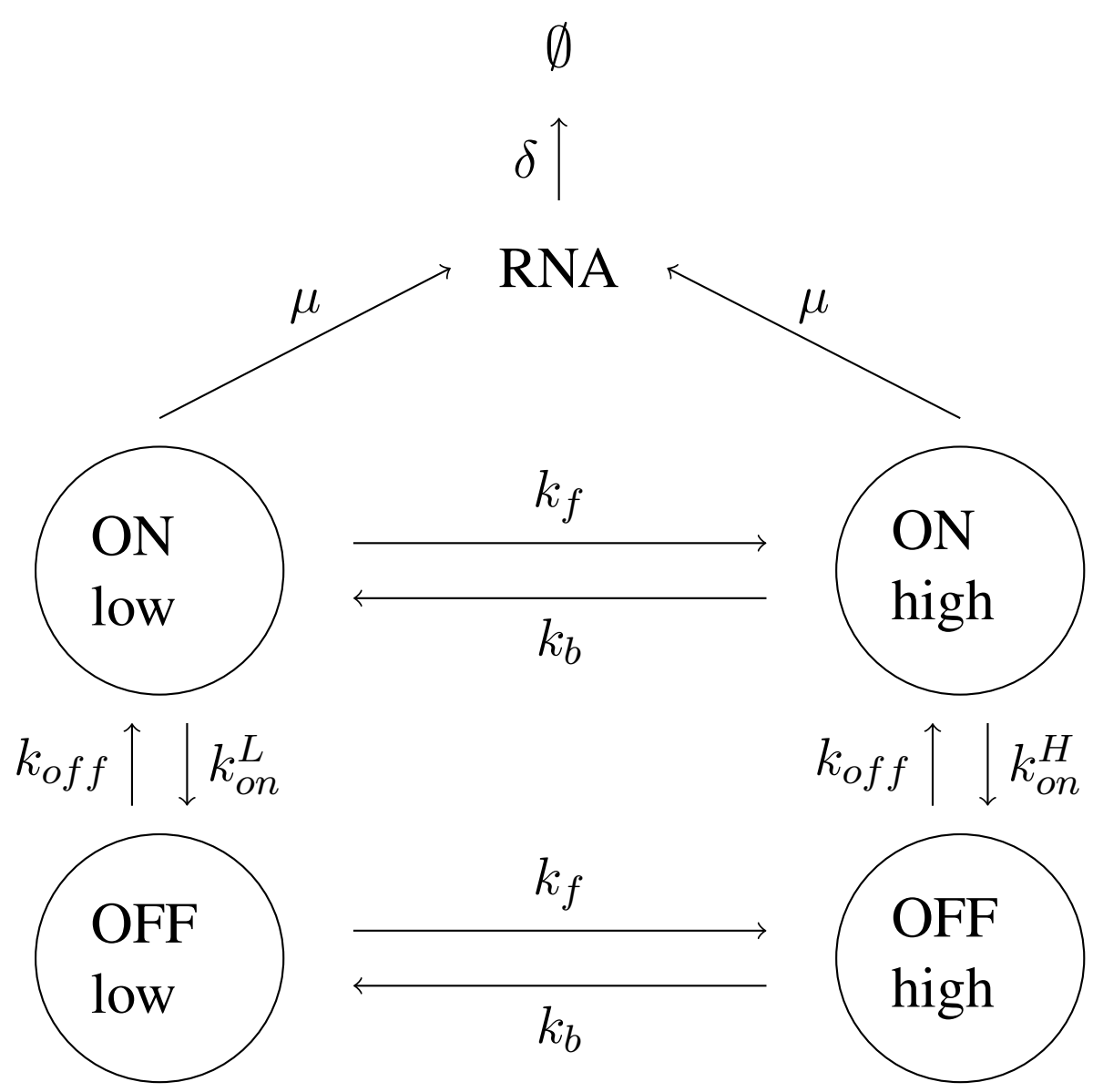

5 Model fitting

The double regime, two state model was fitted to live-cell imaging data in two steps:

1. *Single-Distance Fit*: The model's seven parameters were optimized using data from a single enhancer-promoter distance (5 kb).

2. *Multi-Distance Fit*: For other enhancer-promoter distances, only one parameter was fitted while keeping the remaining parameters fixed at their 5 kb best-fit values.

The following subsections describe the fitting strategy in detail.

5.1 Fitting to a single distance

For the 5 kb enhancer-promoter distance, we fitted simultanuously the model to four experimental observables: (1-2) the survival probabilities of the burst and inter-burst durations, (3) the steady-state probability of observing a burst, and (4) the correlation between consecutive inter-burst durations.

The fitting minimized the weighted sum of squared errors, normalized by the number of data points. To account for both linear and logarithmic scaling in survival probabilities, we minimized a combination of squared errors and squared log errors.

For the optimization, we used a global search approach followed by a local refinement. For the global search, we used the *SAMIN* method (Simulated Annealing) from the Julia package *OptimizationOptimJL*. For local search, we used the *BFGS* method (Broyden–Fletcher–Goldfarb–Shanno algorithm) from the same package.

5.2 Fitting across all distances

For each enhancer-promoter distance (except 5 kb), we fitted the model only to the survival probabilities of the inter-burst durations. Each fit optimized one free parameter at a time, while the remaining six parameters were fixed at their best-fit values from the 5 kb distance. The fitting minimized the squared errors under this constraint.

5.3 Parameter sensitivity analysis

To assess the robustness of our parameter estimates, we performed a parametric bootstrap analysis. We simulated a set of burst trajectories using the double-regime, two-state model with parameters fixed at their best-fit values obtained in Section 5.1. The number and length of simulated trajectories were matched to those of the experimental dataset to ensure comparable statistics. From this simulated dataset, we computed the same observables used in the fitting procedure—namely, the survival probabilities of burst and inter-burst durations, the steady-state burst probability, and the correlation between consecutive inter-burst durations. The double-regime, two-state model was then refitted to these observables, and the resulting best-fit parameter values were recorded. This procedure was repeated 1000 times, and the 99.5% confidence intervals for each parameter were derived from the resulting distributions (see Extended Data Figure 5).

6 Robustness of inter-burst duration correlations and timescales to missed detections of weak bursts

The assumptions of the double regime, two state model are supported by two key observations in the experimental data:

(i) the inter-burst duration distribution exhibits three distinct time scales, and

(ii) there is a positive correlation between consecutive inter-burst durations.

To assess the robustness of these observations to potential detection errors—specifically, the missed identification of weak bursts in our analysis pipeline—we performed the following simulation procedure.

We simulated RNA time traces of the same length as those observed in the experimental data. Within each simulated trace, we identified blocks of consecutive frames containing exactly one mRNA molecule. Each such block was then replaced by a block of zeros with probability

$$p_{miss} \in \{0, 0.1, \dots, 0.9, 1\} \tag{45}$$

From the modified traces, we reconstructed burst tracks by labeling any frame with a positive number of RNA molecules as belonging to a burst. Singlet bursts were then removed following the same procedure as applied to the experimental data. From all the burst tracks, we computed (i) the Kaplan–Meier survival curve of the inter-burst durations, and (ii) the correlation between consecutive inter-burst durations. To determine the number of characteristic time scales in the inter-burst durations, we compared the fits of exponential mixtures containing one, two, three, and four components, as performed for the experimental data. The inferred number of time scales and the inter-burst correlations were then compared with the corresponding ground-truth values analytically derived from the model (see Sections 3.2 and 3.3). The analytically derived time scales with a weight that is below 1% were ignored.

This entire procedure was applied to two generative models:

(1) the three-state model, and

(2) the double regime, two state model.

For each model, we randomly sampled 1000 parameter sets that satisfied the following conditions for the ground-truth inter-burst duration distribution:

1. at least two distinct time scales are present;
2. the weight (probability) of each time scale is at least 10%;
3. the fastest time scale is shorter than 0.9 frames; and
4. any pair of time scales differ by at least a factor of two.

The summary of this analysis is shown in Extended Data Figures 7 and 8. Missing single-RNA bursts leads to underestimation of correlations in the vast majority of parameter sets, even when the error probability is very high (correlations underestimated in 98% of cases with no error, and 88% with 100% error, see Extended Data Fig. 7). Second, error on small bursts also systematically leads to matching or underestimated numbers of inferred time scales (See Extended Data Fig. 8).

References

M. Iosifescu. *Finite Markov Processes and Their Applications*. Wiley, New York, New York, 1980.

B. Munsky and M. Khammash. The finite state projection algorithm for the solution of the chemical master equation. *The Journal of Chemical Physics*, 124(4):044104, 2006. doi: 10.1063/1.2145882.

Gating Strategy MS2 Knock-In

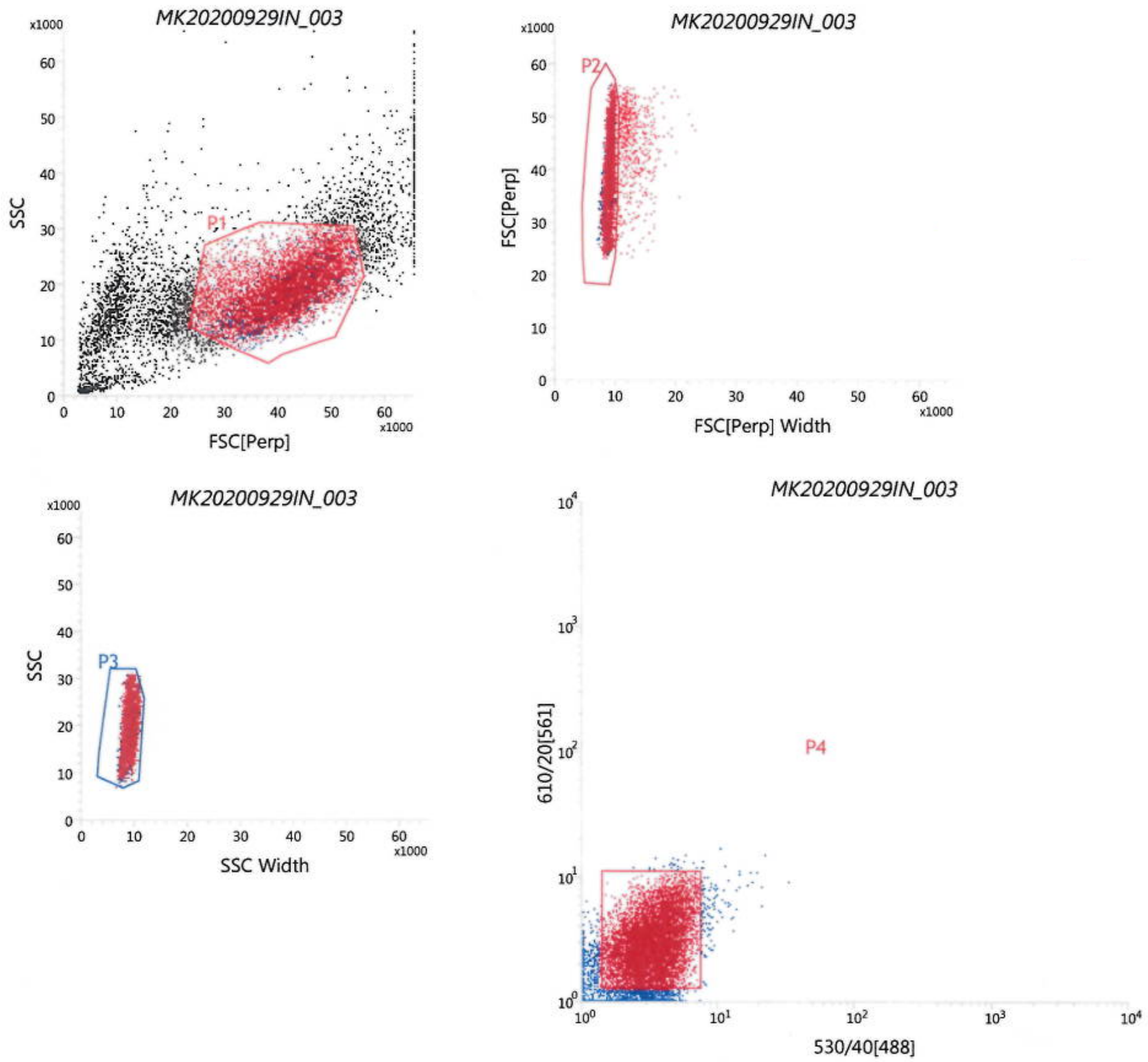

Statistics: *MK20200929IN_003*

Populations	Events	% Total	% Parent	530/40[... Mean	610/20[... Mean
All Events	15,169	100.00%	####	4	3
P1	10,817	71.31%	71.31%	4	3
P2	10,006	65.96%	92.50%	3	3
P3	10,000	65.92%	99.94%	3	3
P4	8,674	57.18%	86.74%	3	3

Gating Strategy:
FSC / SSC to discard big cells with high granularity;
FSC-W / FSC: to discard doublets;
SSC-W / SSC: to discard doublets;
530/40[488] / 610/20[561]: to select suitable autofluorescence cells

Gating Strategy Halo-MCP Knock-in
stained with JF646

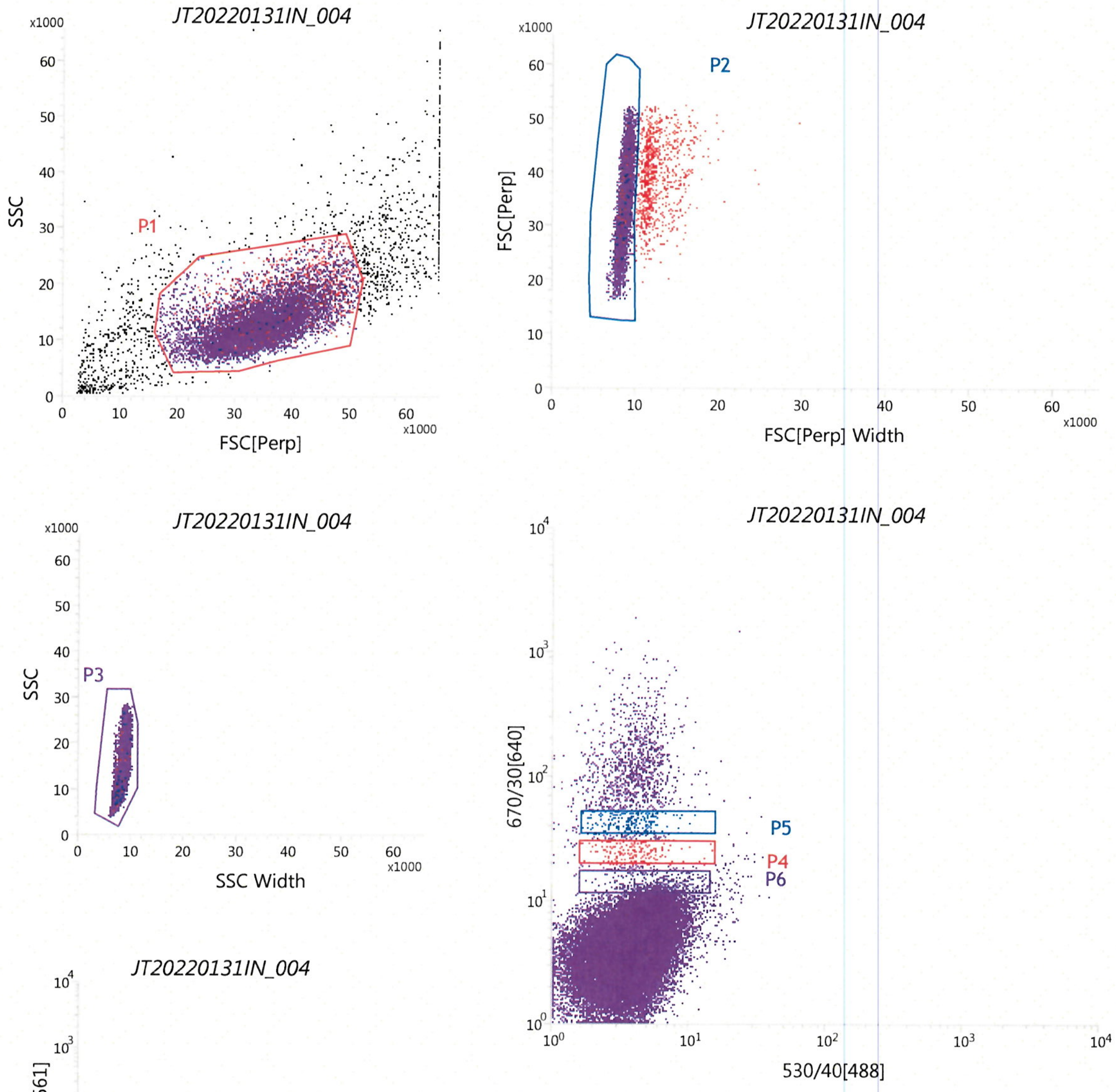

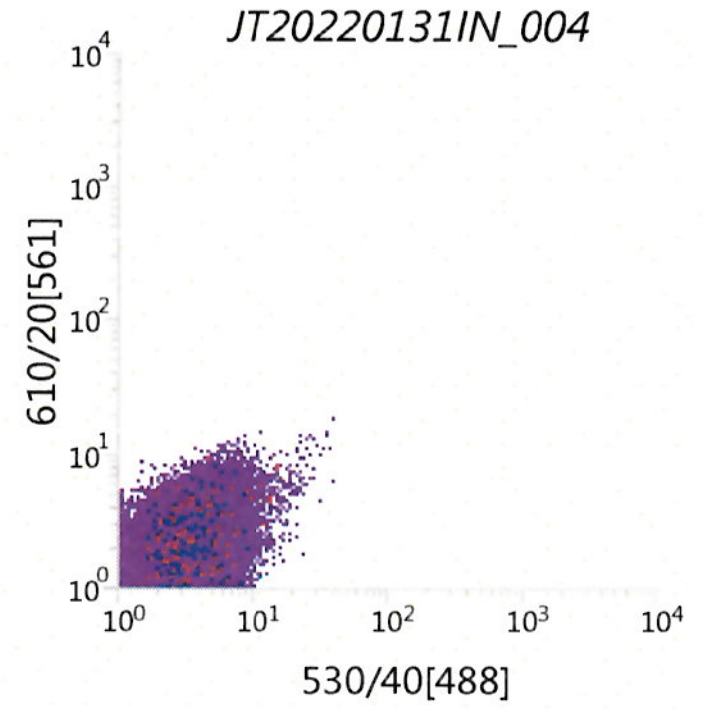

Statistics: *JT20220131IN_004*

Populations	Events	% Total	% Parent	530/40[... Mean	610/20[... Mean
All Events	63,494	100.00%	####	5	3
P1	55,623	87.60%	87.60%	4	3
P2	50,000	78.75%	89.89%	4	3
P3	50,000	78.75%	100.00%	4	3
P4	178	0.28%	0.36%	4	3
P5	202	0.32%	0.40%	4	3
P6	513	0.81%	1.03%	6	3

Gating Strategy:
FSC / SSC to discard big cells with high granularity;
FSC-W / FSC: to discard doublets;
SSC-W / SSC: to discard doublets;
530/40[488] / 610/20[561]: to select suitable Halo-MCP expression, founder cell line originates from P5

Gating Strategy Founder cell line after enhancer mobilisation

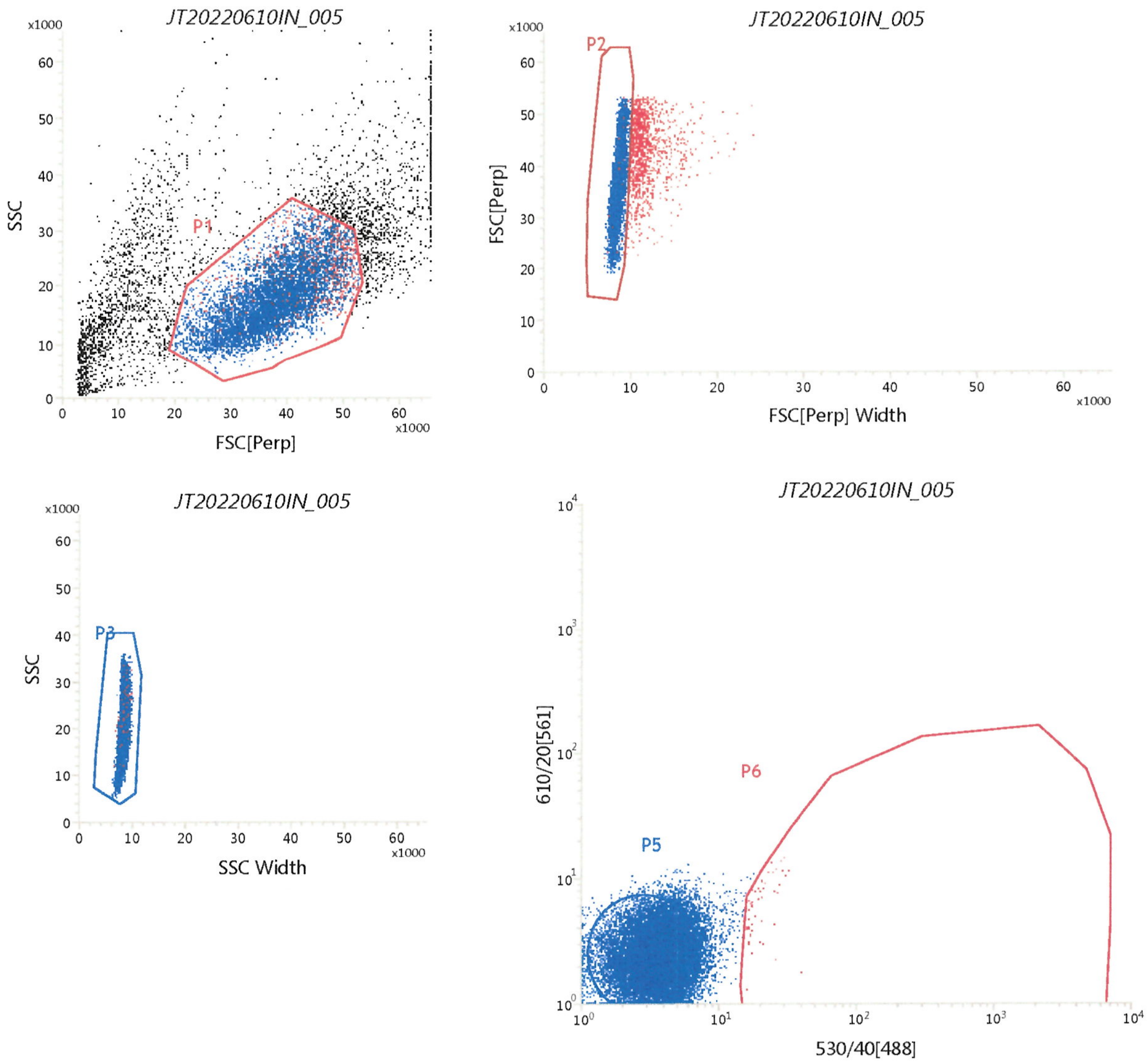

Statistics: *JT20220610IN_005*

Populations	Events	% Total	% Parent	530/40[... Mean	610/20[... Mean
All Events	33,595	100.00%	####	6	3
P1	23,812	70.88%	70.88%	4	3
P2	20,000	59.53%	83.99%	4	3
P3	20,000	59.53%	100.00%	4	3
P5	18,035	53.68%	90.18%	4	3
P6	53	0.16%	0.26%	21	7

Gating Strategy:
FSC / SSC to discard big cells with high granularity;
FSC-W / FSC: to discard doublets;
SSC-W / SSC: to discard doublets;
530/40[488] / 610/20[561]: to discriminate between negative (P5) and GFP positive cells (P4)

Gating Strategy flow cytometry
Measurement GFP expression level of single clones on plate

BD FACSDiva 8.0.1

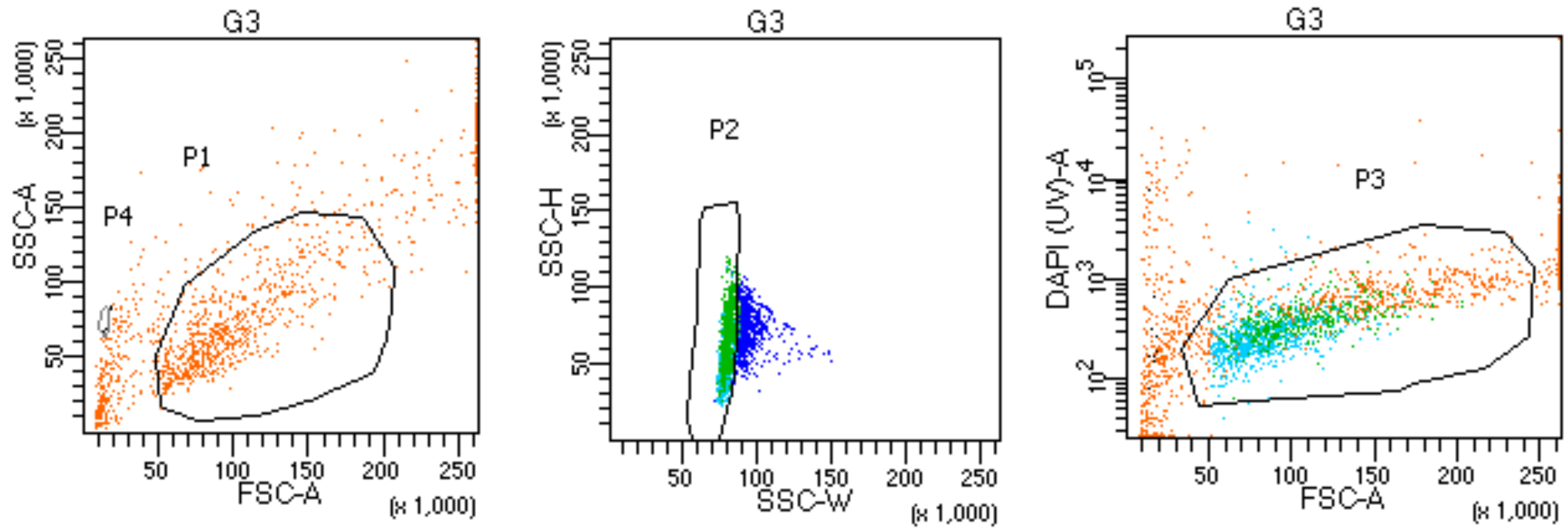

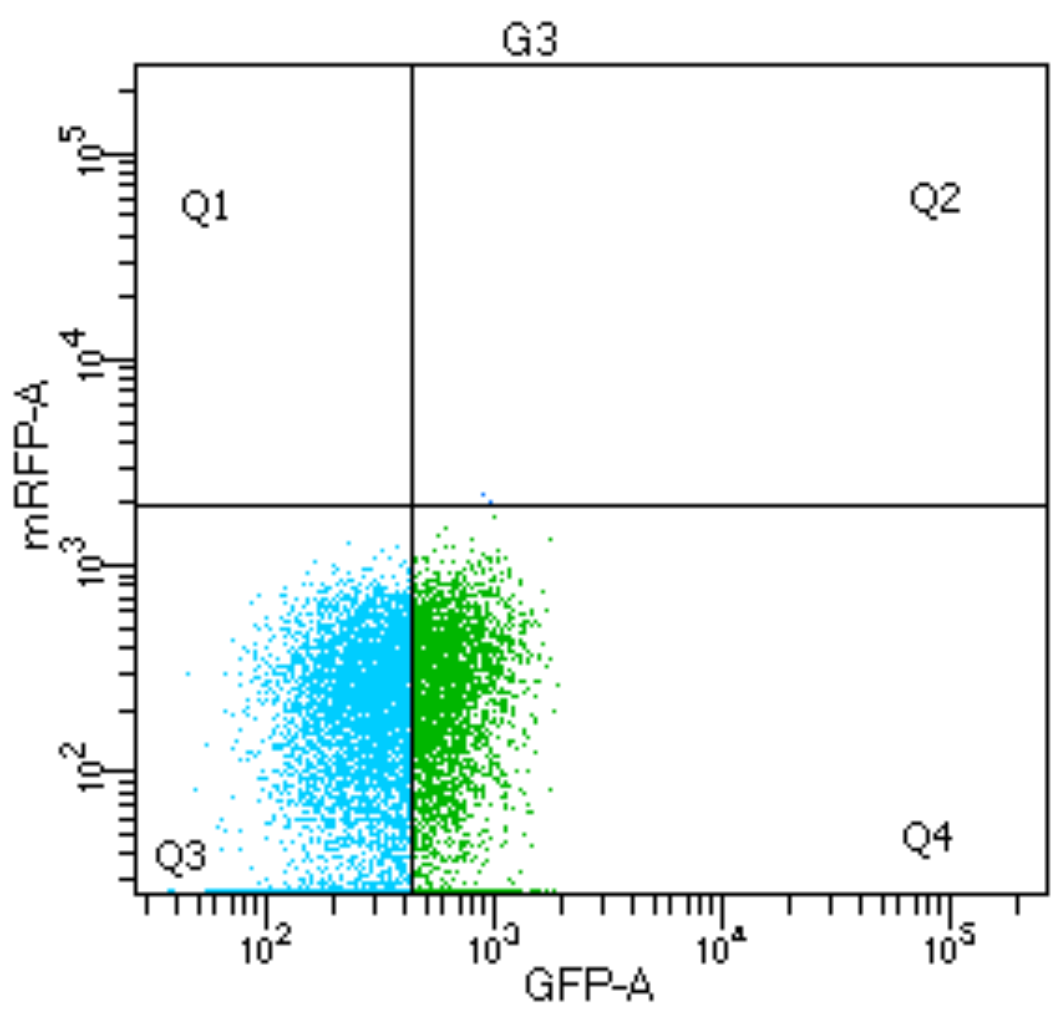

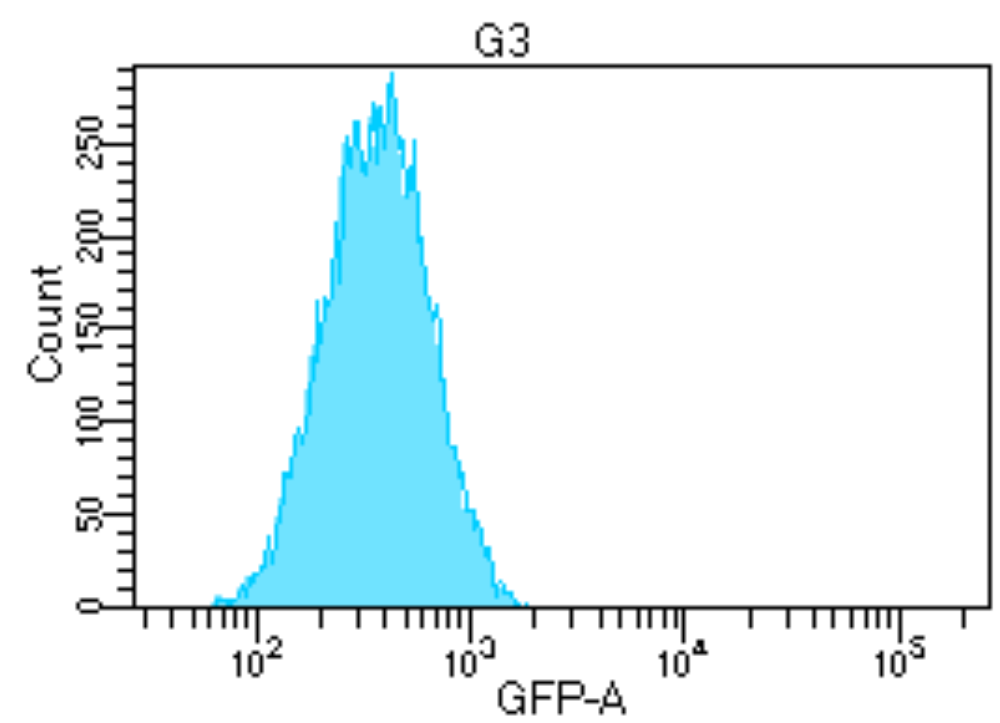

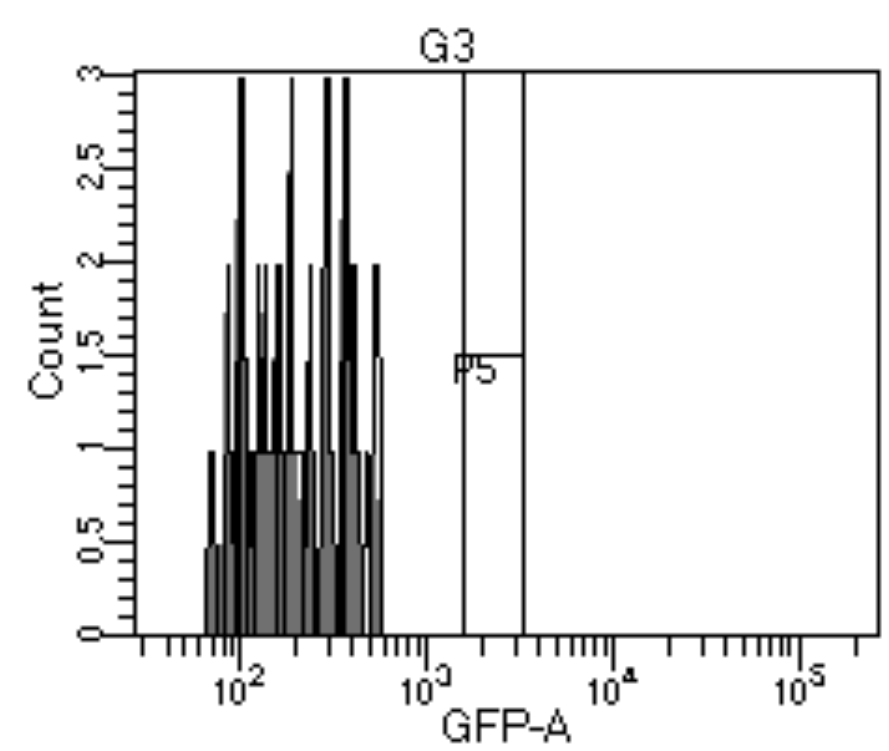

Well Name: G3
SAMPLE ID:

Population	#Events	%Parent	%Total	GFP-A Mean	DsRed-A Mean
All Events	23,694	####	100.0	578	322
P1	13,727	57.9	57.9	468	216
P2	10,785	78.6	45.5	394	182
P3	10,675	99.0	45.1	394	182
Q1	0	0.0	0.0	####	####
Q2	2	0.0	0.0	903	853
Q3	6,987	65.5	29.5	264	152
Q4	3,686	34.5	15.6	641	238
P4	65	0.3	0.3	219	361
P5	0	0.0	0.0	####	####

Well: G3

Population	#Events	%Parent	%Total
All Events	23,694	####	100.0
P1	13,727	57.9	57.9
P2	10,785	78.6	45.5
P3	10,675	99.0	45.1
Q1	0	0.0	0.0
Q2	2	0.0	0.0
Q3	6,987	65.5	29.5
Q4	3,686	34.5	15.6

Gating Strategy:
FSC/SSC to discard big cells with high granularity
SCC-W/SCC-H to discard doublets
FSC-A/Dapi to discard dead cells
GFP/mRFP to visualize GFP expression levels
GFP/histogram to quantify GFP intensity.

Global Sheet1_DAPI

Printed on: Mon Jul 4, 2022 05:07:44 CEST
