# Supplementary material for: Enhancer control of promoter activity and variability via frequency modulation of clustered transcriptional bursts
Source: Nat Genet. 2026 Jul 14;58(8):1967–75. doi: 10.1038/s41588-026-02676-x (PMC13447087; doi:10.1038/s41588-026-02676-x)
Supplement: Supplementary file 2 [file 41588_2026_2676_MOESM2_ESM.pdf]

nature portfolio

Corresponding author(s): Luca Giorgetti

Last updated by author(s): Apr 20, 2026

Reporting Summary

Nature Portfolio wishes to improve the reproducibility of the work that we publish. This form provides structure for consistency and transparency in reporting. For further information on Nature Portfolio policies, see our Editorial Policies and the Editorial Policy Checklist.

Statistics

For all statistical analyses, confirm that the following items are present in the figure legend, table legend, main text, or Methods section.

n/a	Confirmed	
☐	☒	The exact sample size (*n*) for each experimental group/condition, given as a discrete number and unit of measurement
☐	☒	A statement on whether measurements were taken from distinct samples or whether the same sample was measured repeatedly
☒	☐	The statistical test(s) used AND whether they are one- or two-sided<br>*Only common tests should be described solely by name; describe more complex techniques in the Methods section.*
☒	☐	A description of all covariates tested
☒	☐	A description of any assumptions or corrections, such as tests of normality and adjustment for multiple comparisons
☐	☒	A full description of the statistical parameters including central tendency (e.g. means) or other basic estimates (e.g. regression coefficient) AND variation (e.g. standard deviation) or associated estimates of uncertainty (e.g. confidence intervals)
☒	☐	For null hypothesis testing, the test statistic (e.g. *F*, *t*, *r*) with confidence intervals, effect sizes, degrees of freedom and *P* value noted<br>*Give P values as exact values whenever suitable.*
☒	☐	For Bayesian analysis, information on the choice of priors and Markov chain Monte Carlo settings
☒	☐	For hierarchical and complex designs, identification of the appropriate level for tests and full reporting of outcomes
☐	☒	Estimates of effect sizes (e.g. Cohen's *d*, Pearson's *r*), indicating how they were calculated

Our web collection on statistics for biologists contains articles on many of the points above.

Software and code

Policy information about availability of computer code

Data collection	BD LSRII SORP Analyser equipped with BD High Throughput Sampler (HTS) was used for flow cytometry measurements, BD Influx cell sorter was used for the FACS. Live-cell imaging was performed on a Nikon Eclipse Ti-E inverted wide-field microscope with a Total Internal Reflection Microscopy iLAS2 module. Agarose gels were visualized using a Typhoon FLA 9500 scanner (GE Healthcare).
Data analysis	Julia (v.1.10.4). Image analysis code including a detailed list of package versions used is available at https://github.com/fmibasel/ggiorget_ms2analysis .Code used in model inference, analysis, and fitting is available at https://github.com/gregroth/BurstingDynamicsInference.

For manuscripts utilizing custom algorithms or software that are central to the research but not yet described in published literature, software must be made available to editors and reviewers. We strongly encourage code deposition in a community repository (e.g. GitHub). See the Nature Portfolio guidelines for submitting code & software for further information.

Data

Policy information about availability of data

All manuscripts must include a data availability statement. This statement should provide the following information, where applicable:

- Accession codes, unique identifiers, or web links for publicly available datasets
- A description of any restrictions on data availability
- For clinical datasets or third party data, please ensure that the statement adheres to our policy

The raw as well as processed intensity trajectories from imaging data can be found on Zenodo: https://zenodo.org/records/17572944.

Research involving human participants, their data, or biological material

Policy information about studies with human participants or human data. See also policy information about sex, gender (identity/presentation), and sexual orientation and race, ethnicity and racism.

Reporting on sex and gender	no human data were collected.
Reporting on race, ethnicity, or other socially relevant groupings	*Please specify the socially constructed or socially relevant categorization variable(s) used in your manuscript and explain why they were used. Please note that such variables should not be used as proxies for other socially constructed/relevant variables (for example, race or ethnicity should not be used as a proxy for socioeconomic status). Provide clear definitions of the relevant terms used, how they were provided (by the participants/respondents, the researchers, or third parties), and the method(s) used to classify people into the different categories (e.g. self-report, census or administrative data, social media data, etc.) Please provide details about how you controlled for confounding variables in your analyses.*
Population characteristics	*Describe the covariate-relevant population characteristics of the human research participants (e.g. age, genotypic information, past and current diagnosis and treatment categories). If you filled out the behavioural & social sciences study design questions and have nothing to add here, write "See above."*
Recruitment	*Describe how participants were recruited. Outline any potential self-selection bias or other biases that may be present and how these are likely to impact results.*
Ethics oversight	*Identify the organization(s) that approved the study protocol.*

Note that full information on the approval of the study protocol must also be provided in the manuscript.

Field-specific reporting

Please select the one below that is the best fit for your research. If you are not sure, read the appropriate sections before making your selection.

☒ Life sciences ☐ Behavioural & social sciences ☐ Ecological, evolutionary & environmental sciences

For a reference copy of the document with all sections, see nature.com/documents/nr-reporting-summary-flat.pdf

Life sciences study design

All studies must disclose on these points even when the disclosure is negative.

Sample size	For live-cell imaging, 3-7 biological replicates for each cell line of distinct enhancer distance (condition) were performed resulting in on average 1000-2500 tracks/condition analyzed. These tracks originate from ~200-400 cells/condition. We did not apply statistical methods to pre-determine sample size and followed general standard practices in the field. These sample sizes ensure that burst statistics are robust against bootstrapping (see e.g. Fig. 2). For flow cytometry measurements, 3 biological replicates per condition were performed. All other experiments were performed once.
Data exclusions	For live-cell imaging, tracks of active times with more than 20 active frames was manually checked and if wrongly detected excluded. Otherwise, no data was excluded.
Replication	Biological and technical replicates were performed as indicated above. Replicates showed consistent results.
Randomization	Randomization is not applicable to this study as we used only clonal cell lines and no human or animal subjects.
Blinding	Data recording and analysis for live-cell imaging, flow cytometry and Piggybac insertion site mapping were performed in a blinded manner.

Reporting for specific materials, systems and methods

We require information from authors about some types of materials, experimental systems and methods used in many studies. Here, indicate whether each material, system or method listed is relevant to your study. If you are not sure if a list item applies to your research, read the appropriate section before selecting a response.

Materials & experimental systems

n/a	Involved in the study
☒	☐ Antibodies
☐	☒ Eukaryotic cell lines
☒	☐ Palaeontology and archaeology
☒	☐ Animals and other organisms
☒	☐ Clinical data
☒	☐ Dual use research of concern
☒	☐ Plants

Methods

n/a	Involved in the study
☒	☐ ChIP-seq
☐	☒ Flow cytometry
☒	☐ MRI-based neuroimaging

Eukaryotic cell lines

Policy information about cell lines and Sex and Gender in Research

Cell line source(s)	All cell lines are based on E14 mouse embryonic stem cells (mESCs) provided by Edith Heard's laboratory (then at Curie Institute, Paris).
Authentication	Cell lines derived from E14 have been recurrently used by the authors and genotyped multiple times e.g. by whole-genome Oxford Nanopore sequencing), and therefore have not been authenticated.
Mycoplasma contamination	Cells were tested for mycoplasma contamination once a month and no contamination was detected.
Commonly misidentified lines (See ICLAC register)	No commonly misidentified lines were used.

Plants

Seed stocks	*Report on the source of all seed stocks or other plant material used. If applicable, state the seed stock centre and catalogue number. If plant specimens were collected from the field, describe the collection location, date and sampling procedures.*
Novel plant genotypes	*Describe the methods by which all novel plant genotypes were produced. This includes those generated by transgenic approaches, gene editing, chemical/radiation-based mutagenesis and hybridization. For transgenic lines, describe the transformation method, the number of independent lines analyzed and the generation upon which experiments were performed. For gene-edited lines, describe the editor used, the endogenous sequence targeted for editing, the targeting guide RNA sequence (if applicable) and how the editor was applied.*
Authentication	*Describe any authentication procedures for each seed stock used or novel genotype generated. Describe any experiments used to assess the effect of a mutation and, where applicable, how potential secondary effects (e.g. second site T-DNA insertions, mosiacism, off-target gene editing) were examined.*

Flow Cytometry

Plots

Confirm that:

☒ The axis labels state the marker and fluorochrome used (e.g. CD4-FITC).

☒ The axis scales are clearly visible. Include numbers along axes only for bottom left plot of group (a 'group' is an analysis of identical markers).

☒ All plots are contour plots with outliers or pseudocolor plots.

☒ A numerical value for number of cells or percentage (with statistics) is provided.

Methodology

Sample preparation	Flow Cytometry: Cells were harvested with Accutase and re-suspend in E14 medium supplemented with 2i and stained with 500 ng ml–1 DAPI solution (Sigma-Aldrich, D9564). FACS: cells were harvested with Accutase and re-suspend in E14 medium with only 3% of FCS (supplemented with 2i), 100 µg/m primocin (InvivoGen, ant-pm-1) and 10uM ROCK inhibitor (STEMCELL Technologies, Y-27632).
Instrument	BD LSRII SORP Analyser + HTS for enhancer mobilization assay. FACS: BD Influx cell sorter (Becton Dickinson)
Software	Flow Cytometry: BD FACSDiva™ Software. FACS: BD FACS™ Software 1.2.0.142

Cell population abundance	Flow Cytometry: 10000 cells were acquired for enhancer mobilization assay. FACS: single cells sort was performed by sorting 2-6 96-well plates.
Gating strategy	Flow Cytometry gating: FSC/SSC to discard big cells with high granularity; SCC-W/SCC-H to discard doublets; FSC-A/Dapi to discard dead cells; GFP/histogram to quantify GFP intensity. FACS: FSC/SSC to discard big cells with high granularity; FSC-W/FSC: to discard doublets; SSC-W/SSC: to discard doublets; for mobilisation experiments 530/40[488]/610/20[561]: to discriminate between negative and GFP positive cells ; for Halo-Tag insertion, 530/40[488]/670/30[640]: to discriminate between negative and Halo-Tag positive cells.

☒ Tick this box to confirm that a figure exemplifying the gating strategy is provided in the Supplementary Information.
