# Supplementary material for: Enhancer control of promoter activity and variability via frequency modulation of clustered transcriptional bursts
Source: Nat Genet. 2026 Jul 14;58(8):1967–75. doi: 10.1038/s41588-026-02676-x (PMC13447087; doi:10.1038/s41588-026-02676-x)
Supplement: Supplementary file 3 [file 41588_2026_2676_MOESM3_ESM.pdf]

nature portfolio

Peer Review File

Enhancer control of promoter activity and variability via frequency modulation of clustered transcriptional bursts

Corresponding Author: Dr Luca Giorgetti

This file contains all editorial decision letters in order by version, followed by all author rebuttals in order by version.

Version 0:

Reviewer comments:

Reviewer #2

(Remarks to the Author)
The authors use a transposon-based system to scatter an enhancer at random distances from a reporter gene and study the effect of enhancer-promoter contacts on gene expression. This is broadly the same system they employed in their acclaimed earlier study by Zuin et al. However, while the Zuin study was based on population-level measurements of transgene expression, here they have instead modified the system to enable the use of HaloTag-based live imaging. This has made it possible to directly visualise the reporter's transcriptional bursts in real time and study their relationship with the linear enhancer-promoter distance for each integration. The main additional conclusion of the present study is that transcriptional bursts appear clustered, reinforcing the model of different activity regimes at the promoter that are regulated by enhancer contacts – in contrast to earlier models assuming a direct "hit-and-run" relationship between enhancer-promoter contacts and transcriptional bursts. This is a solid and interesting study, and as in the previous work, the formal biophysical modelling of the system is a key strength.

General comments:

1. It would help to present more explicitly the differences between the modelling paradigms in Zuin and this study – beyond what is currently presented in the Discussion (and perhaps address this in other sections as well). It appears that these models are non-mutually exclusive, and there is a slightly different way in which the experimental data were used to fit or validate the model in each case.

2. The authors rightly note that the experiments reported here do not give access to the dynamics of enhancer-promoter distances in 3D (since what is visible by imaging is transcription and not enhancer-promoter looping). And yet, the fact that the study included multiple cell lines with different linear enhancer-promoter distances played a key role in the arguments presented in the Results. This is not necessarily incorrect, but somewhat confusing. Should one expect the nonlinear function to emerge directly from the model? One would certainly expect a plateau at short distances/high contact probability, but perhaps not the threshold? Alternatively, the nonlinear function could be an input to the model, effectively setting the key parameter that varies between enhancers at different genomic distances? Neither seems to be the case here, and the authors do not discuss this.

Minor comments:

1. The Introduction reads as if the study has established a ground truth about all promoters and enhancers, but in the end, this is just a singular case. It would be good to make this clearer.

2. It would be helpful to remind the reader that in this system, promoter contact probabilities at a given distance do not seem to depend on whether an enhancer is incorporated into that region. I also wonder if this is, in fact, a bit unusual, and whether it has any practical implications for the processes dissected here.

3. Fig. 1f: Is there a reason why the error bars aren't shown? Also, would it not make sense to plot the Y axes of 1d and 1f against each other?

4. Burst size and burst amplitude are given in Fig. 2, but not considered again when fitting the model. Why are other

quantities used instead for fitting, but not in Fig. 2?

5. What causes the sudden drop of the red line in Fig. 2d?

6. The authors don't say (unless I missed it) whether it matters that certain tracks were censored for both K-M and bootstrapping. Does this introduce an approximation or not?

7. Supplementary information: Is there a summation over Q missing in the last line of Eq. 34?

8. Removing singlets: Why are these fully removed from the time trace, rather than just treated as a time point without a burst? I assume they are removed because they are likely false positives, but then surely that time point should be counted as part of the gap? Or perhaps I misunderstood what was done and why.

(Remarks on code availability)
The image analysis code is available, but as far as I can tell, not the raw data or code supporting the theory.

Reviewer #3

(Remarks to the Author)
The manuscript by Tunnermann et al. investigates how the genomic location of an enhancer influences transcriptional bursting at a target promoter in mammalian cells, at single-cell resolution. Using a carefully designed synthetic system in mouse embryonic stem cells, the authors mobilise the Sox2 control region (SCR) to different genomic positions relative to a reporter promoter inserted within a neutral TAD. This setup allows them to cleanly isolate the effect of enhancer-promoter distance. They then apply live-cell imaging of MS2-tagged transcripts, combined with rigorous statistical analysis, to characterise promoter burst kinetics across multiple configurations. The results show that transcriptional activity does not occur as isolated stochastic bursts, but rather in temporally clustered patterns, which cannot be well captured by standard two- or three-state models. Instead, the authors propose a regime-switching model, in which the promoter alternates stochastically between low- and high-bursting modes, with the enhancer regulating the entry into the high-activity regime. The study provides a compelling and quantitative dissection of enhancer effects on promoter dynamics. Although it builds on prior studies — including from the same group — which established that enhancer proximity modulates burst frequency, this work goes further by revealing a layered structure in bursting behaviour and showing that enhancer distance governs the likelihood of regime switching. This refinement — from modulating a single rate to switching between burst regimes — provides a more nuanced view of enhancer-promoter communication. The experimental system is exceptionally clean, the data are of high quality, and the modelling is careful and convincing. It is, as such, a significant contribution to our mechanistic understanding of mammalian gene regulation.
Overall, this represents a solid and incremental conceptual advance rather than a paradigm shift. However, the methodological elegance, the clarity of the experimental system, and the depth of analytical modeling may elevate the work to Nature Genetics standards, depending on editorial priorities and the journal's emphasis on technically rigorous but mechanistically nuanced studies.

Comments:
1. Signal quality and measurement robustness
The experimental setup is elegant, using a Sox2 promoter–SCR enhancer pair mobilized into a neutral TAD, with live imaging of MS2-tagged transcripts. However, the manuscript does not provide a quantitative assessment of signal quality. Without a clearer understanding of the signal quality, it is difficult to assess the reliability of the derived burst parameters and the robustness of the downstream modeling. A more detailed analysis of the signal-to-noise ratio, localization precision, and the detection efficiency of transcription sites would strengthen confidence in the burst parameter estimates. Some direct metrics (e.g., per-spot SNR or detection precision) and representative traces would help contextualize the fidelity of burst calling.
2. Burst detection accuracy and control via simulations
The burst detection relies on binary ON/OFF classification based on spot presence, with no modeling of elongation or cleavage kinetics. This is likely acceptable given the MS2 placement in the 3′ UTR and the reported low burst probability (~3%). Still, there is a risk that short ON intervals — particularly those contributing to the fast timescale of OFF intervals — may be misclassified or missed entirely. Misclassification of short ON intervals could lead to an underestimation of burst frequency and an overestimation of the fast timescale of OFF intervals, potentially skewing the interpretation of regime-switching dynamics. To address this, the authors should run their detection and analysis pipeline on simulated time series incorporating realistic measurement noise and known ground-truth kinetics (e.g., two-state, three-state, or two-regime models) to test recovery accuracy and robustness of multi-timescale inference. Given the authors' reliance on precise burst parameter estimation to support their regime-switching model, a rigorous assessment of burst detection accuracy through simulations is essential.
3. Positioning the conceptual advance
The authors' claim that their results 'challenge existing models of mammalian promoter operation' appears overstated and could negatively impact the perception of novelty. A substantial body of literature has already established that enhancers primarily act by tuning burst frequency (e.g., Fukaya et al., Cell 2016; Bartman et al., Mol Cell 2016), and that genomic distance modulates enhancer-promoter communication (e.g., Brückner et al., Science 2023; Zuin et al., Nature 2022). While the current work provides a technically sophisticated and conceptually clarifying validation of established principles in a well-controlled synthetic system, it does not fundamentally revise prevailing models of enhancer action. I strongly recommend moderating the language and framing the contribution as a rigorous and elegant demonstration of layered bursting dynamics, rather than a paradigm shift.

4a. Model interpretation and the role of time-dependent regulation
The proposed double-regime two-state model is a useful phenomenological framework, but it's important to acknowledge that it does not necessarily reject simpler two-state logic. Time-dependent regulation (e.g., TF levels modulating the ON-rate) can also produce similar dynamics. The authors should emphasize that their model demonstrates that regulation can modulate the rate parameters themselves, which may shift across regimes, rather than invalidating simpler models altogether.
4b. The model (Fig. 5a) appears to include a non-equilibrium cycle, violating detailed balance (meaning that the rates of forward and reverse transitions are not equal at equilibrium). If this is the case, the authors should discuss the potential implications and whether active processes (e.g., energy input) might be required to maintain this cycle. Furthermore, they could discuss how time-varying TF concentrations might drive transitions into the high-frequency regime, offering a testable hypothesis for future work.
5. Clarify the relationship between contact frequency and functional activation
The observation that proximity events vastly outnumber functional transitions is a key insight that deserves greater emphasis. This suggests that most contacts are either non-productive or that multiple contacts (or long-lived ones) are required to push the promoter into a transcriptionally active regime. A clearer articulation of this point is critical for connecting the observed kinetics to possible molecular mechanisms. The authors should explicitly discuss whether they envision regime transitions as being triggered by rare single contacts with low success probability or as the result of a ratcheting mechanism involving multiple transient encounters.
6. Consider alternative explanations for burst clustering
While the clustered burst pattern is nicely captured by the two-regime model, alternative interpretations should at least be acknowledged. For instance, it is possible that clustering reflects a form of promoter memory, transient chromatin accessibility, or Pol II recycling — none of which are explicitly modeled here. The authors might briefly discuss whether such mechanisms could, in principle, produce similar statistics, and whether their model captures this as an effective coarse-graining. Clarifying this point would help situate their results within broader discussions on the molecular origins of bursting.
7. Quantify model support and parameter identifiability
The manuscript includes AIC/BIC values in Supplementary Table S1 to justify model selection, which is helpful. However, a brief discussion of parameter identifiability (e.g., through confidence intervals or sensitivity analysis) would further strengthen confidence that the model structure is both necessary and well constrained by the data.
8. Expand on the biological implications of transcriptional variability
The link between enhancer-promoter distance and cell-to-cell variability in burst number is intriguing and potentially important. The authors suggest that local enhancers may support more uniform output, while distal ones introduce heterogeneity. This raises interesting questions: do genes that require robust, tightly controlled expression tend to have proximal enhancers, while genes involved in bet-hedging or fate diversification rely more on distal regulation? Even a brief comment pointing toward this broader regulatory logic would give more depth to the manuscript's implications for genome organization and gene function.

(Remarks on code availability)

Decision Letter:

25th Jun 2025

Dear Dr Giorgetti,

apologize for the delay in providing these reviews.

Your Article, "Enhancer control of promoter activity and variability via frequency modulation of clustered transcriptional bursts" has now been seen by 2 referees. You will see from their comments below that while they find your work of interest, some points are raised. We are interested in the possibility of publishing your study in Nature Genetics, but would like to consider your response to these concerns in the form of a revised manuscript before we make a final decision on publication.

Reviewer #1 thinks the study is solid and interesting. They ask to compare the current model to the previous one in Zuin et al, and further discuss some critical points.

Reviewer #2 thinks your study represents a solid but incremental advance however, the elegance of the method and depth of the analytical modelling elevate the work. They ask to position the conceptual advance, to strengthen confidence in the burst parameter estimates with further analyses and to provide a rigorous assessment of burst detection accuracy through simulations.

To guide the scope of the revisions, the editors discuss the referee reports in detail within the team, including with the chief editor, with a view to identifying key priorities that should be addressed in revision and sometimes overruling referee requests that are deemed beyond the scope of the current study. In this case, we please ask you to address Reviewers' comments in full. Please do not hesitate to get in touch if you would like to discuss these issues further.

We therefore invite you to revise your manuscript taking into account all reviewer and editor comments. Please highlight all

changes in the manuscript text file. At this stage we will need you to upload a copy of the manuscript in MS Word .docx or similar editable format.

We are committed to providing a fair and constructive peer-review process. Do not hesitate to contact us if there are specific requests from the reviewers that you believe are technically impossible or unlikely to yield a meaningful outcome.

When revising your manuscript:

*1) Include a "Response to referees" document detailing, point-by-point, how you addressed each referee comment. If no action was taken to address a point, you must provide a compelling argument. This response will be sent back to the referees along with the revised manuscript.

*2) If you have not done so already please begin to revise your manuscript so that it conforms to our Article format instructions, available
here.
Refer also to any guidelines provided in this letter.

*3) Include a revised version of any required Reporting Summary: https://www.nature.com/documents/nr-reporting-summary.pdf
It will be available to referees (and, potentially, statisticians) to aid in their evaluation if the manuscript goes back for peer review.
A revised checklist is essential for re-review of the paper.

Please be aware of our guidelines on digital image standards.

EXTENDED DATA FIGURES

When re-submitting your manuscript, please ensure that any supplementary figures and tables that are crucial to the manuscript's conclusions are converted into Extended Data figures and tables to increase visibility of these data. Extended Data figures and tables are online-only (present in the online PDF and full-text HTML versions of the paper), peer-reviewed display items that provide essential background to the article but are not included in the main article due to space constraints. A maximum of ten Extended Data display items (figures and tables) is permitted.

Please use the link below to submit your revised manuscript and related files:

Link Redacted

Note: This URL links to your confidential home page and associated information about manuscripts you may have submitted, or that you are reviewing for us. If you wish to forward this email to co-authors, please delete the link to your homepage.

We hope to receive your revised manuscript within four to eight weeks. If you cannot send it within this time, please let us know.

Please do not hesitate to contact me if you have any questions or would like to discuss these revisions further.

Nature Genetics is committed to improving transparency in authorship. As part of our efforts in this direction, we are now requesting that all authors identified as 'corresponding author' on published papers create and link their Open Researcher and Contributor Identifier (ORCID) with their account on the Manuscript Tracking System (MTS), prior to acceptance. ORCID helps the scientific community achieve unambiguous attribution of all scholarly contributions. You can create and link your ORCID from the home page of the MTS by clicking on 'Modify my Springer Nature account'. For more information please visit please visit www.springernature.com/orcid.

We look forward to seeing the revised manuscript and thank you for the opportunity to review your work.

Sincerely,
Chiara

Chiara Anania, PhD
Associate Editor
Nature Genetics
https://orcid.org/0000-0003-1549-4157

Referee expertise:

Referee #1: 3D genomics, transcription regulation

Referee #2: biophysics, gene regulation

Reviewers' Comments:

Reviewer #2 (Remarks to the Author):

The authors use a transposon-based system to scatter an enhancer at random distances from a reporter gene and study the effect of enhancer-promoter contacts on gene expression. This is broadly the same system they employed in their acclaimed earlier study by Zuin et al. However, while the Zuin study was based on population-level measurements of transgene expression, here they have instead modified the system to enable the use of HaloTag-based live imaging. This has made it possible to directly visualise the reporter's transcriptional bursts in real time and study their relationship with the linear enhancer-promoter distance for each integration. The main additional conclusion of the present study is that transcriptional bursts appear clustered, reinforcing the model of different activity regimes at the promoter that are regulated by enhancer contacts – in contrast to earlier models assuming a direct "hit-and-run" relationship between enhancer-promoter contacts and transcriptional bursts. This is a solid and interesting study, and as in the previous work, the formal biophysical modelling of the system is a key strength.

General comments:

1. It would help to present more explicitly the differences between the modelling paradigms in Zuin and this study – beyond what is currently presented in the Discussion (and perhaps address this in other sections as well). It appears that these models are non-mutually exclusive, and there is a slightly different way in which the experimental data were used to fit or validate the model in each case.

2. The authors rightly note that the experiments reported here do not give access to the dynamics of enhancer-promoter distances in 3D (since what is visible by imaging is transcription and not enhancer-promoter looping). And yet, the fact that the study included multiple cell lines with different linear enhancer-promoter distances played a key role in the arguments presented in the Results. This is not necessarily incorrect, but somewhat confusing. Should one expect the nonlinear function to emerge directly from the model? One would certainly expect a plateau at short distances/high contact probability, but perhaps not the threshold? Alternatively, the nonlinear function could be an input to the model, effectively setting the key parameter that varies between enhancers at different genomic distances? Neither seems to be the case here, and the authors do not discuss this.

Minor comments:

1. The Introduction reads as if the study has established a ground truth about all promoters and enhancers, but in the end, this is just a singular case. It would be good to make this clearer.

2. It would be helpful to remind the reader that in this system, promoter contact probabilities at a given distance do not seem to depend on whether an enhancer is incorporated into that region. I also wonder if this is, in fact, a bit unusual, and whether it has any practical implications for the processes dissected here.

3. Fig. 1f: Is there a reason why the error bars aren't shown? Also, would it not make sense to plot the Y axes of 1d and 1f against each other?

4. Burst size and burst amplitude are given in Fig. 2, but not considered again when fitting the model. Why are other quantities used instead for fitting, but not in Fig. 2?

5. What causes the sudden drop of the red line in Fig. 2d?

6. The authors don't say (unless I missed it) whether it matters that certain tracks were censored for both K-M and bootstrapping. Does this introduce an approximation or not?

7. Supplementary information: Is there a summation over Q missing in the last line of Eq. 34?

8. Removing singlets: Why are these fully removed from the time trace, rather than just treated as a time point without a burst? I assume they are removed because they are likely false positives, but then surely that time point should be counted as part of the gap? Or perhaps I misunderstood what was done and why.

Reviewer #2 (Remarks on code availability):

The image analysis code is available, but as far as I can tell, not the raw data or code supporting the theory.

Reviewer #3 (Remarks to the Author):

The manuscript by Tunnermann et al. investigates how the genomic location of an enhancer influences transcriptional bursting at a target promoter in mammalian cells, at single-cell resolution. Using a carefully designed synthetic system in mouse embryonic stem cells, the authors mobilise the Sox2 control region (SCR) to different genomic positions relative to a reporter promoter inserted within a neutral TAD. This setup allows them to cleanly isolate the effect of enhancer-promoter distance. They then apply live-cell imaging of MS2-tagged transcripts, combined with rigorous statistical analysis, to characterise promoter burst kinetics across multiple configurations. The results show that transcriptional activity does not occur as isolated stochastic bursts, but rather in temporally clustered patterns, which cannot be well captured by standard two- or three-state models. Instead, the authors propose a regime-switching model, in which the promoter alternates stochastically between low- and high-bursting modes, with the enhancer regulating the entry into the high-activity regime. The study provides a compelling and quantitative dissection of enhancer effects on promoter dynamics. Although it builds on prior studies — including from the same group — which established that enhancer proximity modulates burst frequency, this work goes further by revealing a layered structure in bursting behaviour and showing that enhancer distance governs the likelihood of regime switching. This refinement — from modulating a single rate to switching between burst regimes — provides a more nuanced view of enhancer-promoter communication. The experimental system is exceptionally clean, the data are of high quality, and the modelling is careful and convincing. It is, as such, a significant contribution to our mechanistic understanding of mammalian gene regulation.
Overall, this represents a solid and incremental conceptual advance rather than a paradigm shift. However, the methodological elegance, the clarity of the experimental system, and the depth of analytical modeling may elevate the work to Nature Genetics standards, depending on editorial priorities and the journal's emphasis on technically rigorous but mechanistically nuanced studies.

Comments:
1. Signal quality and measurement robustness
The experimental setup is elegant, using a Sox2 promoter–SCR enhancer pair mobilized into a neutral TAD, with live imaging of MS2-tagged transcripts. However, the manuscript does not provide a quantitative assessment of signal quality. Without a clearer understanding of the signal quality, it is difficult to assess the reliability of the derived burst parameters and the robustness of the downstream modeling. A more detailed analysis of the signal-to-noise ratio, localization precision, and the detection efficiency of transcription sites would strengthen confidence in the burst parameter estimates. Some direct metrics (e.g., per-spot SNR or detection precision) and representative traces would help contextualize the fidelity of burst calling.
2. Burst detection accuracy and control via simulations
The burst detection relies on binary ON/OFF classification based on spot presence, with no modeling of elongation or cleavage kinetics. This is likely acceptable given the MS2 placement in the 3′ UTR and the reported low burst probability (~3%). Still, there is a risk that short ON intervals — particularly those contributing to the fast timescale of OFF intervals — may be misclassified or missed entirely. Misclassification of short ON intervals could lead to an underestimation of burst frequency and an overestimation of the fast timescale of OFF intervals, potentially skewing the interpretation of regime-switching dynamics. To address this, the authors should run their detection and analysis pipeline on simulated time series incorporating realistic measurement noise and known ground-truth kinetics (e.g., two-state, three-state, or two-regime models) to test recovery accuracy and robustness of multi-timescale inference. Given the authors' reliance on precise burst parameter estimation to support their regime-switching model, a rigorous assessment of burst detection accuracy through simulations is essential.
3. Positioning the conceptual advance
The authors' claim that their results 'challenge existing models of mammalian promoter operation' appears overstated and could negatively impact the perception of novelty. A substantial body of literature has already established that enhancers primarily act by tuning burst frequency (e.g., Fukaya et al., Cell 2016; Bartman et al., Mol Cell 2016), and that genomic distance modulates enhancer-promoter communication (e.g., Brückner et al., Science 2023; Zuin et al., Nature 2022). While the current work provides a technically sophisticated and conceptually clarifying validation of established principles in a well-controlled synthetic system, it does not fundamentally revise prevailing models of enhancer action. I strongly recommend moderating the language and framing the contribution as a rigorous and elegant demonstration of layered bursting dynamics, rather than a paradigm shift.
4a. Model interpretation and the role of time-dependent regulation
The proposed double-regime two-state model is a useful phenomenological framework, but it's important to acknowledge that it does not necessarily reject simpler two-state logic. Time-dependent regulation (e.g., TF levels modulating the ON-rate) can also produce similar dynamics. The authors should emphasize that their model demonstrates that regulation can modulate the rate parameters themselves, which may shift across regimes, rather than invalidating simpler models altogether.
4b. The model (Fig. 5a) appears to include a non-equilibrium cycle, violating detailed balance (meaning that the rates of forward and reverse transitions are not equal at equilibrium). If this is the case, the authors should discuss the potential implications and whether active processes (e.g., energy input) might be required to maintain this cycle. Furthermore, they could discuss how time-varying TF concentrations might drive transitions into the high-frequency regime, offering a testable hypothesis for future work.
5. Clarify the relationship between contact frequency and functional activation
The observation that proximity events vastly outnumber functional transitions is a key insight that deserves greater emphasis. This suggests that most contacts are either non-productive or that multiple contacts (or long-lived ones) are required to push the promoter into a transcriptionally active regime. A clearer articulation of this point is critical for connecting the observed kinetics to possible molecular mechanisms. The authors should explicitly discuss whether they envision regime transitions as being triggered by rare single contacts with low success probability or as the result of a ratcheting mechanism involving multiple transient encounters.

6. Consider alternative explanations for burst clustering
While the clustered burst pattern is nicely captured by the two-regime model, alternative interpretations should at least be acknowledged. For instance, it is possible that clustering reflects a form of promoter memory, transient chromatin accessibility, or Pol II recycling — none of which are explicitly modeled here. The authors might briefly discuss whether such mechanisms could, in principle, produce similar statistics, and whether their model captures this as an effective coarse-graining. Clarifying this point would help situate their results within broader discussions on the molecular origins of bursting.
7. Quantify model support and parameter identifiability
The manuscript includes AIC/BIC values in Supplementary Table S1 to justify model selection, which is helpful. However, a brief discussion of parameter identifiability (e.g., through confidence intervals or sensitivity analysis) would further strengthen confidence that the model structure is both necessary and well constrained by the data.
8. Expand on the biological implications of transcriptional variability
The link between enhancer-promoter distance and cell-to-cell variability in burst number is intriguing and potentially important. The authors suggest that local enhancers may support more uniform output, while distal ones introduce heterogeneity. This raises interesting questions: do genes that require robust, tightly controlled expression tend to have proximal enhancers, while genes involved in bet-hedging or fate diversification rely more on distal regulation? Even a brief comment pointing toward this broader regulatory logic would give more depth to the manuscript's implications for genome organization and gene function.

Version 1:

Reviewer comments:

Reviewer #2

(Remarks to the Author)
I thank the authors for addressing my comments in full and have no further queries.

(Remarks on code availability)
I have not reviewed the code but I thank the authors for making it available, along with minimal example data and a README file with instructions.

Reviewer #3

(Remarks to the Author)
I thank the authors for their detailed and thoughtful response, which substantially improves the manuscript. The additions are significant and well aligned with the core points raised in my initial review. In particular, the new figures quantifying signal quality (S2–S3), the simulation-based validation of the burst-calling pipeline (S8), and the extended modeling discussion—including parameter identifiability, contact frequency vs. functional activation, and the thermodynamic implications of their non-equilibrium cycle—are all welcome and well-executed. While the bibliography now includes several relevant studies, the prioritization of more peripheral references over prior work that is more directly connected to the core experimental and conceptual contributions remains puzzling and should be reconsidered. I also appreciate the revised framing of the conceptual advance, which now avoids overstating novelty and positions the work as a mechanistic refinement of existing models. The authors acknowledge alternative interpretations of burst clustering and clarify the phenomenological nature of their two-regime model, bringing appropriate nuance to their conclusions. Overall, the manuscript now presents a rigorous and carefully interpreted study of enhancer-dependent transcriptional bursting in a well-controlled system. I find my concerns satisfactorily addressed and support publication.

(Remarks on code availability)

Decision Letter:

Our ref: NG-A68640R

29th Dec 2025

Dear Luca,

Thank you for submitting your revised manuscript entitled "Enhancer control of promoter activity and variability via frequency modulation of clustered transcriptional bursts" (NG-A68640R). It has been seen by the original referees and their comments are below. The reviewers find that the paper has improved in revision, and therefore we'll be happy in principle to publish it in Nature Genetics, pending minor revisions to comply with our editorial and formatting guidelines.

The current version of your manuscript is in a PDF format. Please email us (natgen@us.nature.com) a copy of the file in an editable format (Microsoft Word) - we can not proceed with PDFs at this stage.

We will then be performing detailed checks on your paper and will send you a checklist detailing our editorial and formatting requirements soon. Please do not upload the final materials and make any revisions until you receive this additional

information from us.

Thank you again for your interest in Nature Genetics. Please do not hesitate to contact me if you have any questions.

Congratulations!

Sincerely,

Tiago

Tiago Faial, PhD
Chief Editor
Nature Genetics
https://orcid.org/0000-0003-0864-1200

Reviewer #2 (Remarks to the Author):

I thank the authors for addressing my comments in full and have no further queries.

Reviewer #2 (Remarks on code availability):

I have not reviewed the code but I thank the authors for making it available, along with minimal example data and a README file with instructions.

Reviewer #3 (Remarks to the Author):

I thank the authors for their detailed and thoughtful response, which substantially improves the manuscript. The additions are significant and well aligned with the core points raised in my initial review. In particular, the new figures quantifying signal quality (S2–S3), the simulation-based validation of the burst-calling pipeline (S8), and the extended modeling discussion—including parameter identifiability, contact frequency vs. functional activation, and the thermodynamic implications of their non-equilibrium cycle—are all welcome and well-executed. While the bibliography now includes several relevant studies, the prioritization of more peripheral references over prior work that is more directly connected to the core experimental and conceptual contributions remains puzzling and should be reconsidered. I also appreciate the revised framing of the conceptual advance, which now avoids overstating novelty and positions the work as a mechanistic refinement of existing models. The authors acknowledge alternative interpretations of burst clustering and clarify the phenomenological nature of their two-regime model, bringing appropriate nuance to their conclusions. Overall, the manuscript now presents a rigorous and carefully interpreted study of enhancer-dependent transcriptional bursting in a well-controlled system. I find my concerns satisfactorily addressed and support publication.

Open Access This Peer Review File is licensed under a Creative Commons Attribution 4.0 International License, which permits use, sharing, adaptation, distribution and reproduction in any medium or format, as long as you give appropriate credit to the original author(s) and the source, provide a link to the Creative Commons license, and indicate if changes were made.
In cases where reviewers are anonymous, credit should be given to 'Anonymous Referee' and the source.
The images or other third party material in this Peer Review File are included in the article's Creative Commons license, unless indicated otherwise in a credit line to the material. If material is not included in the article's Creative Commons license and your intended use is not permitted by statutory regulation or exceeds the permitted use, you will need to obtain permission directly from the copyright holder.
To view a copy of this license, visit https://creativecommons.org/licenses/by/4.0/

We thank the referees for their very positive and constructive criticism. We have addressed all comments through new analyses, additional controls, and substantial revisions of the text. All modified or newly added sentences are highlighted in red in the revised manuscript for clarity.

In brief, the main revisions are as follows:

1. We performed new analyses to exclude the possibility that model selection is biased by the misdetection of weak or short bursts (**new Extended Data Figs. 7 and 8**).
2. We conducted a comprehensive sensitivity analysis to assess the robustness of key model parameters (**modified Extended Data Fig. 5**).
3. We included SNR distributions and more representative MS2 traces in the new **Extended Data Fig. 2**, and included precision/recall analysis for spot detection against manually annotated data in the text.
4. We expanded the comparison between the current model and that inferred in our previous work (Zuin, Roth et al., 2022), hopefully providing a clearer perspective on their relationship and differences.
5. We revised sections that previously overstated the novelty of our model, aiming to position our findings more accurately within the broader context of the field and the ongoing debate on the origins of transcriptional bursts.
6. Inspired by the Reviewers' suggestions, we expanded the discussion of potential mechanistic underpinnings of the coarse-grained model behavior, incorporating references to recent work from our group and others that offer improved quantitative insights into chromosomal looping dynamics.

Point-by-point responses to reviewers's comments:

Reviewer #2 (Remarks to the Author):

The authors use a transposon-based system to scatter an enhancer at random distances from a reporter gene and study the effect of enhancer-promoter contacts on gene expression. This is broadly the same system they employed in their acclaimed earlier study by Zuin et al. However, while the Zuin study was based on population-level measurements of transgene expression, here they have instead modified the system to enable the use of HaloTag-based live imaging. This has made it possible to directly visualise the reporter's transcriptional bursts in real time and study their relationship with the linear enhancer-promoter distance for each integration. The main additional conclusion of the present study is that transcriptional bursts appear clustered, reinforcing the model of different activity regimes at the promoter that are regulated by enhancer contacts – in contrast to earlier models assuming a direct "hit-and-run" relationship

between enhancer-promoter contacts and transcriptional bursts. This is a solid and interesting study, and as in the previous work, the formal biophysical modelling of the system is a key strength.

We thank the referee for their appreciation of our work.

General comments:

1. It would help to present more explicitly the differences between the modelling paradigms in Zuin and this study – beyond what is currently presented in the Discussion (and perhaps address this in other sections as well). It appears that these models are non-mutually exclusive, and there is a slightly different way in which the experimental data were used to fit or validate the model in each case.

We agree with the Reviewer that this was a bit confusing in the initial version of the manuscript. We now expanded the Results and Discussion sections by adding new paragraphs that hopefully give a better perspective of the similarities and differences between the two models (page 9 and 10 in the manuscript).
As now explained in the manuscript, the structure of the two models is indeed very similar (both have two regimes with two states, which differ in their ON rates). In our previous work, flow-cytometry and smFISH data led us to assume that when the enhancer is in close genomic proximity with the promoter (and hence in *physical* proximity nearly 100% of the time), the promoter worked 100% of the time in the high-frequency two-state regime. However, the new live-cell imaging data presented in this study reveals that this is not the case, because even when the enhancer is as close as ~5k, the promoter displays three silent timescales that are not compatible with two-state operation. This finding forced us to drop the assumption that continuous association with the enhancer causes continuous operation in the high-frequency regime. We thus resorted to a simpler model that is agnostic as to how mechanistically enhancer-promoter encounters are translated into regime changes. We hope that the new text clarifies this. We note that in response to the Reviewer's second point below as well as comments by Reviewer #3, we also expanded the discussion of how promoter transitions might arise from enhancer-promoter encounters in the light of the most recent measurements of chromosome looping dynamics (page 11).

2. The authors rightly note that the experiments reported here do not give access to the dynamics of enhancer-promoter distances in 3D (since what is visible by imaging is transcription and not enhancer-promoter looping). And yet, the fact that the study included multiple cell lines with different linear enhancer-promoter distances played a key role in the arguments presented in the Results. This is not necessarily incorrect, but somewhat confusing. Should one expect the nonlinear function to emerge directly from the model?

One would certainly expect a plateau at short distances/high contact probability, but perhaps not the threshold? Alternatively, the nonlinear function could be an input to the model, effectively setting the key parameter that varies between enhancers at different genomic distances? Neither seems to be the case here, and the authors do not discuss this.

We apologize about the confusion regarding the role of enhancer-promoter interactions and the origins of nonlinearity. We hope that the improved Results and Discussion help to clarify this important point. The simpler model we present in this study is completely agnostic to the mechanistic link between enhancer-promoter interactions and promoter operation, and as such is not expected to predict the nonlinear relationship between transcription and enhancer-promoter contact probabilities. As shown in Fig. 6 however, our analysis shows that the transition rate from the low to the high regime changes as a function of where the enhancer is located, and thus as a function of their contact probability. As we now better explain in the text and report in the **new Extended Data Fig. 5g**, this relationship is nonlinear, as expected from the observation that the burst frequency changes nonlinearly with contact probabilities. While the model does not provide a mechanistic explanation for how this happens, we now expanded the Discussion to speculate on possible mechanistic explanations: either through consecutive short-lived contacts, as we previously proposed, or via a subset of longer-lived and potentially cohesin-driven encounters - which we recently discovered and reported in another manuscript now posted on bioRxiv (Ubertini, Louafi et al, DOI:10.1101/2025.09.24.678119).

Minor comments:

1. The Introduction reads as if the study has established a ground truth about all promoters and enhancers, but in the end, this is just a singular case. It would be good to make this clearer.

The revised text should help clarify that our results concern the Sox2-SCR pair, and not necessarily every other enhancer and promoter.

2. It would be helpful to remind the reader that in this system, promoter contact probabilities at a given distance do not seem to depend on whether an enhancer is incorporated into that region. I also wonder if this is, in fact, a bit unusual, and whether it has any practical implications for the processes dissected here.

We now explicitly mention this point in the Results section (page 4). We would like to emphasize, however, that in Zuin, Roth et al., 2022 we demonstrated that even if Capture-C measurements underestimated site-specific interactions involving the SCR and the

Sox2 promoter, the resulting relationship would remain nonlinear. Specifically, we showed that artificially scaling the measured contact probabilities at enhancer–promoter insertion sites (either by additive or multiplicative factors of varying magnitude) led only to quantitative shifts in the curve, without altering its fundamental nonlinearity.

3. Fig. 1f: Is there a reason why the error bars aren't shown? Also, would it not make sense to plot the Y axes of 1d and 1f against each other?

Error bars in Fig. 1f are absent as each point represents the probability to observe a burst over the entire dataset (all movies and MS2 trajectories considered together). We now provide an inset in Fig. 1f with the requested scatter plot with the correlation between population-averaged mean EGFP levels and burst probabilities.

4. Burst size and burst amplitude are given in Fig. 2, but not considered again when fitting the model. Why are other quantities used instead for fitting, but not in Fig. 2?

This is because contrary to burst and inter-burst durations, which are expressed in units of time, burst amplitudes and sizes have arbitrary (fluorescence) units. As also explained below to point 2 raised by Reviewer #3, conversion of such arbitrary units into absolute numbers of RNA would require calibration of transcription site (TS) intensities with smRNA-FISH measurements. Unfortunately, in this experimental system bursts are small due to the low initiation rate, and their detection is highly confounded by the signals emanating from single nuclear mRNAs. On the other hand, burst and inter-burst durations have absolute time units and can be quantitatively fitted without any further assumptions.

5. What causes the sudden drop of the red line in Fig. 2d?

Empirical survival curves are inherently discrete, taking values only at durations actually observed in the data. Consequently, when long events are rare, their small sample size can introduce substantial point-to-point variability in the estimated curve.

6. The authors don't say (unless I missed it) whether it matters that certain tracks were censored for both K-M and bootstrapping. Does this introduce an approximation or not?

The Reviewer is right that censoring can affect the estimation of survival probabilities. The Kaplan-Meier (K-M) estimator is a non-parametric method that accounts for censoring when estimating survival probabilities from lifetime data (see a similar discussion in a recent study from the Hansen lab: Gabriele et al: DOI: 10.1126/science.abn6583). We therefore used the K-M estimator to compute survival probabilities for burst and inter-burst durations. In contrast, the K-M estimator is not suitable for non-lifetime data such as burst amplitude or burst size. Although censoring

events might also occur for these observables, in our data most bursts were fully observed because they are short-lived (2924 out of 2973). We therefore ignored censoring in these cases and computed the empirical survival probabilities directly. We hope that this clarifies this issue, and have now added one sentence in the Methods section to better explain the rationale.

7. Supplementary information: Is there a summation over Q missing in the last line of Eq. 34?

Thank you for pointing that out, that is really well spotted. We have now corrected it.

8. Removing singlets: Why are these fully removed from the time trace, rather than just treated as a time point without a burst? I assume they are removed because they are likely false positives, but then surely that time point should be counted as part of the gap? Or perhaps I misunderstood what was done and why.

Apologies for the lack of clarity - indeed, the singlets were replaced by time points without a burst (i.e. 1 -> 0). We have now clarified this point in the methods section (page 17).

Reviewer #2 (Remarks on code availability):

The image analysis code is available, but as far as I can tell, not the raw data or code supporting the theory.
We now provide the raw MS2 trajectories, the nuclear and local background values, and processed trajectories at https://zenodo.org/records/17572944. Code can be now found at https://github.com/gregroth/BurstingDynamicsInference. We have updated the Data Availability statement accordingly.

Reviewer #3 (Remarks to the Author):

The manuscript by Tunnermann et al. investigates how the genomic location of an enhancer influences transcriptional bursting at a target promoter in mammalian cells, at single-cell resolution. Using a carefully designed synthetic system in mouse embryonic stem cells, the authors mobilise the Sox2 control region (SCR) to different genomic positions relative to a reporter promoter inserted within a neutral TAD. This setup allows them to cleanly isolate the effect of enhancer-promoter distance. They then apply live-cell imaging of MS2-tagged transcripts, combined with rigorous statistical analysis, to characterise promoter burst kinetics across multiple configurations. The results show that transcriptional activity does not occur as isolated stochastic bursts, but rather in

temporally clustered patterns, which cannot be well captured by standard two- or three-state models. Instead, the authors propose a regime-switching model, in which the promoter alternates stochastically between low- and high-bursting modes, with the enhancer regulating the entry into the high-activity regime.

The study provides a compelling and quantitative dissection of enhancer effects on promoter dynamics. Although it builds on prior studies — including from the same group — which established that enhancer proximity modulates burst frequency, this work goes further by revealing a layered structure in bursting behaviour and showing that enhancer distance governs the likelihood of regime switching. This refinement — from modulating a single rate to switching between burst regimes — provides a more nuanced view of enhancer-promoter communication. The experimental system is exceptionally clean, the data are of high quality, and the modelling is careful and convincing. It is, as such, a significant contribution to our mechanistic understanding of mammalian gene regulation. Overall, this represents a solid and incremental conceptual advance rather than a paradigm shift. However, the methodological elegance, the clarity of the experimental system, and the depth of analytical modeling may elevate the work to Nature Genetics standards, depending on editorial priorities and the journal's emphasis on technically rigorous but mechanistically nuanced studies.

We thank the Reviewer for their appreciation of our work.

Comments:

1. Signal quality and measurement robustness

The experimental setup is elegant, using a Sox2 promoter–SCR enhancer pair mobilized into a neutral TAD, with live imaging of MS2-tagged transcripts. However, the manuscript does not provide a quantitative assessment of signal quality. Without a clearer understanding of the signal quality, it is difficult to assess the reliability of the derived burst parameters and the robustness of the downstream modeling. A more detailed analysis of the signal-to-noise ratio, localization precision, and the detection efficiency of transcription sites would strengthen confidence in the burst parameter estimates. Some direct metrics (e.g., per-spot SNR or detection precision) and representative traces would help contextualize the fidelity of burst calling.

We agree with the Reviewer that this is an important point that was not adequately addressed in the initial version of the manuscript. We now report the per-spot SNR distribution and example signal traces in the new **Extended Data Fig. 2**, and precision/recall against manually annotated data in the text.

2. Burst detection accuracy and control via simulations

The burst detection relies on binary ON/OFF classification based on spot presence, with no modeling of elongation or cleavage kinetics. This is likely acceptable given the MS2 placement in the 3′ UTR and the reported low burst probability (~3%). Still, there is a risk that short ON intervals — particularly those contributing to the fast timescale of OFF intervals — may be misclassified or missed entirely. Misclassification of short ON intervals could lead to an underestimation of burst frequency and an overestimation of the fast timescale of OFF intervals, potentially skewing the interpretation of regime-switching dynamics. To address this, the authors should run their detection and analysis pipeline on simulated time series incorporating realistic measurement noise and known ground-truth kinetics (e.g., two-state, three-state, or two-regime models) to test recovery accuracy and robustness of multi-timescale inference. Given the authors' reliance on precise burst parameter estimation to support their regime-switching model, a rigorous assessment of burst detection accuracy through simulations is essential.

We agree that this is a very important point. We did our best to implement the Reviewer's request to run our detection and analysis pipeline on artificial images with similar noise levels and statistics and signal-to-noise ratios, where time-resolved signal intensities were generated by alternative model scenarios. However, converting absolute numbers of RNA in the model into fluorescence intensities requires an absolute calibration constant. We initially thought about using smFISH for calibration, similar to Chen et al., DOI: 10.1038/s41594-025-01615-4 but realized that in our experimental setup this is affected by one major caveat: TSS intensities are very small due to the small burst size and mostly overlap those of single mRNA signals; so unfortunately in the absence of an intron, this makes TSS identification extremely tricky. We thus believe that this approach would be affected by extremely large levels of uncertainty and would be dependent on arbitrary parameter choices.
However, we took the Reviewer's comment to heart and have now addressed their concerns using a different strategy. The proposed double regime, two-state model was selected to satisfy two key observations in the data: (i) the inter-burst duration distribution exhibits three distinct timescales, and (ii) there is a positive correlation between consecutive inter-burst durations. Therefore, it is imperative to test that the inference of the three timescales and the detection of correlation are poorly sensitive to potential missed detections of short or weak bursts. To address this question, we simulated RNA time traces from two different models: a standard three-state model, and our two-state, two-regime model. To simulate the effect of missed detection of weak bursts, we then artificially forced a fraction (ranging from 0 to 100%) of consecutive frames containing exactly one RNA to instead contain zero RNA. In order to match the statistics of our data, the number of tracks and their length were matched to the experimental dataset. We then processed the simulated and binarized burst traces as we did for the experimental ones. The inferred number of time scales and inter-burst correlations were then compared with

the corresponding ground-truth that we analytically derived from the models. This comparison was performed for 1000 different parameter sets (see Supplementary Information for more details).
As reported in the new Extended Data Fig. 7 and 8, this analysis strongly supports our conclusion: First, missing single-RNA bursts leads to *underestimation* of correlations in the vast majority of parameter sets, even when the error probability is very high (correlations underestimated in 98% of cases with no error, and 88% with 100% error, see Extended Data Fig. 7). Second, error on small bursts also systematically leads to matching or *underestimated* numbers of inferred time scales (See Extended Data Fig. 8). We hope that these analyses convincingly show that the detection of positive inter-burst correlations and of three distinct timescales is robust to mis- and missed detection of small bursts. These results are now also reported in a new paragraph at the end of the Results section. Detailed methods are also included in the Supplementary Information.

3. Positioning the conceptual advance
The authors' claim that their results 'challenge existing models of mammalian promoter operation' appears overstated and could negatively impact the perception of novelty. A substantial body of literature has already established that enhancers primarily act by tuning burst frequency (e.g., Fukaya et al., Cell 2016; Bartman et al., Mol Cell 2016), and that genomic distance modulates enhancer-promoter communication (e.g., Brückner et al., Science 2023; Zuin et al., Nature 2022). While the current work provides a technically sophisticated and conceptually clarifying validation of established principles in a well-controlled synthetic system, it does not fundamentally revise prevailing models of enhancer action. I strongly recommend moderating the language and framing the contribution as a rigorous and elegant demonstration of layered bursting dynamics, rather than a paradigm shift.

We agree that our model does not challenge existing models of enhancer action, but rather expands them by revealing new properties of bursting dynamics. We apologize if our initial manuscript sounded too pompous in this regard and now substantially toned down sentences that might convey excessive novelty (notably in the Abstract and discussion).

4a. Model interpretation and the role of time-dependent regulation
The proposed double-regime two-state model is a useful phenomenological framework, but it's important to acknowledge that it does not necessarily reject simpler two-state logic. Time-dependent regulation (e.g., TF levels modulating the ON-rate) can also produce similar dynamics. The authors should emphasize that their model demonstrates that

regulation can modulate the rate parameters themselves, which may shift across regimes, rather than invalidating simpler models altogether.

We agree with the Reviewer on this interesting comment! We now added a sentence to account for this possibility in the Discussion (page 11). The same sentence should also allow the reader to make the link with (possibly time-dependent) transcription factor concentrations and potential mechanisms for the out-of-equilibrium cycle (see following comment).

4b. The model (Fig. 5a) appears to include a non-equilibrium cycle, violating detailed balance (meaning that the rates of forward and reverse transitions are not equal at equilibrium). If this is the case, the authors should discuss the potential implications and whether active processes (e.g., energy input) might be required to maintain this cycle. Furthermore, they could discuss how time-varying TF concentrations might drive transitions into the high-frequency regime, offering a testable hypothesis for future work.

The Reviewer is right again: indeed, the two-state, two-regime model violates the detailed balance and is hence not at thermodynamic equilibrium. We added notes at page 9 (Results) and page 11 (Discussion) to describe this aspect of the model. In response to the Reviewer's comment, and also to address comment n.2 by Reviewer #2, we now have also modified the Discussion to acknowledge that the rare low-to-high promoter regime transitions might be associated with the long-lived and mainly cohesin-driven chromosomal encounters that we recently reported in another manuscript (Ubertini, Louafi et al, bioRviv 2025). These encounters depend on the ATP-driven loop extrusion activity of cohesin and hence are energy-dependent, which we speculate might contribute to the maintenance of the non-equilibrium cycle.

5. Clarify the relationship between contact frequency and functional activation
The observation that proximity events vastly outnumber functional transitions is a key insight that deserves greater emphasis. This suggests that most contacts are either non-productive or that multiple contacts (or long-lived ones) are required to push the promoter into a transcriptionally active regime. A clearer articulation of this point is critical for connecting the observed kinetics to possible molecular mechanisms. The authors should explicitly discuss whether they envision regime transitions as being triggered by rare single contacts with low success probability or as the result of a ratcheting mechanism involving multiple transient encounters.

This point is now addressed in the same paragraph in the Discussion (page 11).

6. Consider alternative explanations for burst clustering

While the clustered burst pattern is nicely captured by the two-regime model, alternative interpretations should at least be acknowledged. For instance, it is possible that clustering reflects a form of promoter memory, transient chromatin accessibility, or Pol II recycling — none of which are explicitly modeled here. The authors might briefly discuss whether such mechanisms could, in principle, produce similar statistics, and whether their model captures this as an effective coarse-graining. Clarifying this point would help situate their results within broader discussions on the molecular origins of bursting.

In the model that we inferred in this work, each cluster of bursts corresponds to an ON period, whereas single bursts within the cluster correspond to independent initiation events (since the initiation rate is slow, initiation events can be separated by apparent "inactive" periods with the promoter being still in the ON state). The burst properties within a cluster are thus the same in the two promoter regimes, but the frequency at which a cluster occurs differs. Perhaps the Reviewer refers to the maintenance of the high-frequency promoter regime, for which we have no mechanistic explanation, but which we can speculate might indeed be associated with some kind of molecular memory (possibly chromatin modifications, accessibility, PolII recycling). We have now further expanded the Discussion to briefly speculate about the potential origins of such 'memory' (page 11).

7. Quantify model support and parameter identifiability
The manuscript includes AIC/BIC values in Supplementary Table S1 to justify model selection, which is helpful. However, a brief discussion of parameter identifiability (e.g., through confidence intervals or sensitivity analysis) would further strengthen confidence that the model structure is both necessary and well constrained by the data.

We have now included parameter sensitivity analysis in the form of a parametric bootstrap from which we derived 99.5% confidence intervals (CI). This should provide a conservative estimate of the robustness of the fit. The CIs are now reported in the Results section in the main text (page 8) and listed in the parameter tables in the updated version of Extended Data Fig. 5. The bootstrap procedure is described in detail in the Supplementary Information file.

8. Expand on the biological implications of transcriptional variability
The link between enhancer-promoter distance and cell-to-cell variability in burst number is intriguing and potentially important. The authors suggest that local enhancers may support more uniform output, while distal ones introduce heterogeneity. This raises interesting questions: do genes that require robust, tightly controlled expression tend to have proximal enhancers, while genes involved in bet-hedging or fate diversification rely more on distal regulation? Even a brief comment pointing toward this broader regulatory

logic would give more depth to the manuscript's implications for genome organization and gene function.

We agree that this is an intriguing observation, and we indeed hint at this possibility in the manuscript, also mentioning that the Phillips-Cremins (Beagan et al., DOI:10.1038/s41593-020-0634-6) and Merkenschlager labs (Calderon et al., DOI:10.7554/eLife.76539) previously reported that in neurons, early-response genes - which need to be activated robustly and rapidly in all cells - tend to be regulated by short-range enhancers, whereas late-response genes tend to have longer-range regulatory sequences that depend on cohesin (page 12). Although it would be exciting to see if similar patterns apply to other inducible systems (e.g. the inflammatory or hypoxia responses), we are not aware of any additional systematic correlations of this kind.
